# A Novel Framework for Phenotyping Children With Suspected or Confirmed Infection for Future Biomarker Studies

**DOI:** 10.3389/fped.2021.688272

**Published:** 2021-07-28

**Authors:** Ruud G. Nijman, Rianne Oostenbrink, Henriette A. Moll, Climent Casals-Pascual, Ulrich von Both, Aubrey Cunnington, Tisham De, Irini Eleftheriou, Marieke Emonts, Colin Fink, Michiel van der Flier, Ronald de Groot, Myrsini Kaforou, Benno Kohlmaier, Taco W. Kuijpers, Emma Lim, Ian K. Maconochie, Stephane Paulus, Federico Martinon-Torres, Marko Pokorn, Sam T. Romaine, Irene Rivero Calle, Luregn J. Schlapbach, Frank J. Smit, Maria Tsolia, Effua Usuf, Victoria J. Wright, Shunmay Yeung, Dace Zavadska, Werner Zenz, Michael Levin, Jethro A. Herberg, Enitan D. Carrol, The PERFORM consortium (Personalized Risk assessment in febrile children to optimize Real-life Management across the European Union)

**Affiliations:** ^1^Section of Pediatric Infectious Disease, Department of Infectious Disease, Faculty of Medicine, Imperial College of Science, Technology and Medicine, London, United Kingdom; ^2^Department of Pediatric Accident and Emergency, Imperial College NHS Healthcare Trust, London, United Kingdom; ^3^Department of General Pediatrics, Erasmus MC-Sophia Children's Hospital, Rotterdam, Netherlands; ^4^Nuffield Department of Medicine, Wellcome Trust Centre for Human Genetics, University of Oxford, Oxford, United Kingdom; ^5^Department of Clinical Microbiology, Hospital Clínic de Barcelona, Biomedical Diagnostic Centre, Barcelona, Spain; ^6^ISGlobal, Barcelona Institute for Global Health, Barcelona, Spain; ^7^Division of Pediatric Infectious Diseases, Dr. von Hauner Children's Hospital, University Hospital, Ludwig-Maximilians-University, Munich, Germany; ^8^German Centre for Infection Research, DZIF, Partner Site Munich, Munich, Germany; ^9^Second Department of Pediatrics, P. and A. Kyriakou Children's Hospital, National and Kapodistrian University of Athens, Athens, Greece; ^10^Pediatric Immunology, Infectious Diseases and Allergy Department, Great North Children's Hospital, Newcastle upon Tyne Hospitals Foundation Trust, Newcastle upon Tyne, United Kingdom; ^11^Population Health Sciences Institute, Newcastle University, Newcastle upon Tyne, United Kingdom; ^12^National Institute for Health Research Newcastle Biomedical Research Centre Based at Newcastle upon Tyne Hospitals NHS Trust, Newcastle University, Newcastle upon Tyne, United Kingdom; ^13^Micropathology Ltd., Warwick, United Kingdom; ^14^Section Pediatric Infectious Diseases, Laboratory of Medical Immunology, Pediatric Infectious Diseases and Immunology, Radboud Centre for Infectious Diseases, Amalia Children's Hospital, Radboud Institute for Molecular Life Sciences, Radboud University Medical Centre, Nijmegen, Netherlands; ^15^Pediatric Infectious Diseases and Immunology, Wilhelmina Children's Hospital, University Medical Centre Utrecht, Utrecht, Netherlands; ^16^Department of General Pediatrics, Medical University of Graz, Graz, Austria; ^17^Department of Pediatric Immunology, Rheumatology and Infectious Diseases, Amsterdam University Medical Center, Location Academic Medical Centre, University of Amsterdam, Amsterdam, Netherlands; ^18^Landsteiner Laboratory at the Amsterdam Medical Centre, Sanquin Research Institute, University of Amsterdam, Amsterdam, Netherlands; ^19^Department of Pediatrics, Children's Hospital, John Radcliffe, University of Oxford, Level 2, Oxford, United Kingdom; ^20^Institute of Infection and Global Health, University of Liverpool, Liverpool, United Kingdom; ^21^Genetics, Vaccines, Infections and Pediatrics Research Group, Hospital Clínico Universitario de Santiago de Compostela, Santiago de Compostela, Spain; ^22^Department of Infectious Diseases, University Medical Centre Ljubljana, Univerzitetni Klinični Centre, Ljubljana, Slovenia; ^23^Department of Infectious Diseases and Epidemiology, Faculty of Medicine, University of Ljubljana, Ljubljana, Slovenia; ^24^Department of Intensive Care and Neonatology, Children's Research Center, University Children's Hospital Zurich, Zurich, Switzerland; ^25^Child Health Research Centre, The University of Queensland, Brisbane, QLD, Australia; ^26^Department of Pediatrics, Maasstad Hospital, Rotterdam, Netherlands; ^27^Child Survival, Medical Research Council: The Gambia Unit, Fajara, Gambia; ^28^Faculty of Tropical and Infectious Disease, London School of Hygiene and Tropical Medicine, London, United Kingdom; ^29^Department of Pediatrics, Children Clinical University Hospital, Rigas Stradina Universitāte, Riga, Latvia; ^30^Alder Hey Children's NHS Foundation Trust, Liverpool, United Kingdom; ^31^Liverpool Health Partners, Liverpool, United Kingdom

**Keywords:** serious bacterial infection, children, biomarkers, sepsis, clinical phenotypes

## Abstract

**Background:** The limited diagnostic accuracy of biomarkers in children at risk of a serious bacterial infection (SBI) might be due to the imperfect reference standard of SBI. We aimed to evaluate the diagnostic performance of a new classification algorithm for biomarker discovery in children at risk of SBI.

**Methods:** We used data from five previously published, prospective observational biomarker discovery studies, which included patients aged 0– <16 years: the Alder Hey emergency department (*n* = 1,120), Alder Hey pediatric intensive care unit (*n* = 355), Erasmus emergency department (*n* = 1,993), Maasstad emergency department (*n* = 714) and St. Mary's hospital (*n* = 200) cohorts. Biomarkers including procalcitonin (PCT) (4 cohorts), neutrophil gelatinase-associated lipocalin-2 (NGAL) (3 cohorts) and resistin (2 cohorts) were compared for their ability to classify patients according to current standards (dichotomous classification of SBI vs. non-SBI), vs. a proposed PERFORM classification algorithm that assign patients to one of eleven categories. These categories were based on clinical phenotype, test outcomes and C-reactive protein level and accounted for the uncertainty of final diagnosis in many febrile children. The success of the biomarkers was measured by the Area under the receiver operating Curves (AUCs) when they were used individually or in combination.

**Results:** Using the new PERFORM classification system, patients with clinically confident bacterial diagnosis (“definite bacterial” category) had significantly higher levels of PCT, NGAL and resistin compared with those with a clinically confident viral diagnosis (“definite viral” category). Patients with diagnostic uncertainty had biomarker concentrations that varied across the spectrum. AUCs were higher for classification of “definite bacterial” vs. “definite viral” following the PERFORM algorithm than using the “SBI” vs. “non-SBI” classification; summary AUC for PCT was 0.77 (95% CI 0.72–0.82) vs. 0.70 (95% CI 0.65–0.75); for NGAL this was 0.80 (95% CI 0.69–0.91) vs. 0.70 (95% CI 0.58–0.81); for resistin this was 0.68 (95% CI 0.61–0.75) vs. 0.64 (0.58–0.69) The three biomarkers combined had summary AUC of 0.83 (0.77–0.89) for “definite bacterial” vs. “definite viral” infections and 0.71 (0.67–0.74) for “SBI” vs. “non-SBI.”

**Conclusion:** Biomarkers of bacterial infection were strongly associated with the diagnostic categories using the PERFORM classification system in five independent cohorts. Our proposed algorithm provides a novel framework for phenotyping children with suspected or confirmed infection for future biomarker studies.

## Introduction

**Figure 1:**
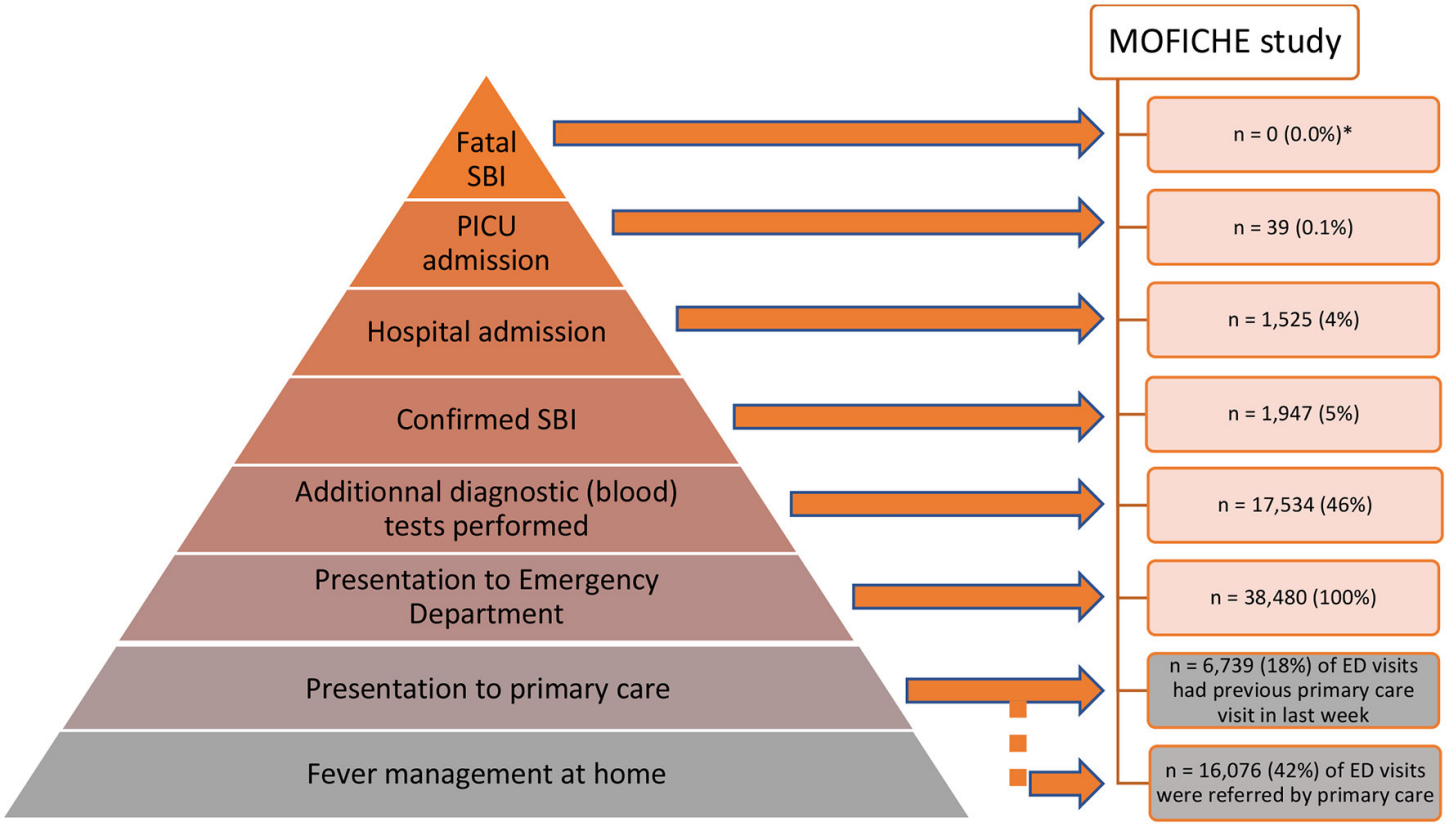
Child with fever: patient journey in order of likely outcome. A small proportion of children presenting to the ED with a febrile illness have a confirmed Serious Bacterial Infection (SBI), and of these a smaller number require admission to hospital or PICU, as shown in the pyramid as a percentage of the total number of febrile children in ED. The data were collected in the MOFICHE study (Management and Outcome of Fever in Children in Europe, *n* = 38,480) as part of the EU Horizon 2020-funded PERFORM study (Personalized Risk assessment in Febrile illness to Optimize Real-life Management across the European Union, www.perform2020.org). The MOFICHE study was an observational study in twelve EDs in eight different European countries [Austria, Germany, Greece, Latvia, the Netherlands (*n* = 3), Spain, Slovenia and the United Kingdom (*n* = 3)], which recorded clinical data on consecutive children with febrile illness in 2017–2018 (78). There were no fatal cases of SBI in the MOFICHE study, but 1 case of fatal viral gastro-enteritis; PICU admission with SBI: 39 (25%) out of total of 158 PICU admissions; hospital admission with SBI: 1,947 (20%) out of total of 9.893 admissions; *the MOFICHE study reflects death in ED, not overall mortality. ED, emergency department; SBI, serious bacterial infection; PICU, pediatric intensive care unit.

Amongst the many children presenting with febrile illness to healthcare, a minority have serious bacterial infections (SBI), and of those, only the rare few are admitted to intensive care units or have a fatal outcome (Figure 1). Clinical signs and symptoms alone lack the ability to reliably differentiate between children with viral and bacterial infection, and accurate biomarkers are urgently needed (1). SBIs are still one of the leading causes of childhood mortality and morbidity in both high income as well as low and middle income countries (2, 3). The increasing global burden of antimicrobial resistance has amplified the need for improved diagnostics, and there has been an emphasis on the need for “rule-out” tests for bacterial infection, so that antibiotic treatment can be reserved for those needing treatment, irrespective of the presence of coincident viral infection. In addition, sepsis campaigns have promoted early identification of children at risk of sepsis and the early escalation of care, including prompt administration of broad spectrum antibiotics, making improved biomarkers to guide management even more urgent (4–8). In high income countries, an increasing proportion of children who present to the emergency department (ED) with SBI have pre-existing co-morbidities, which can make the diagnosis more challenging (9, 10). The economic impact of diagnostic uncertainty when managing pediatric febrile illness is significant, with the precautionary use of antibiotics being associated with increased costs (11).

With effective antivirals emerging or in the pipeline for common and important viral illnesses [including coronavirus and Respiratory Syncytial Virus (RSV)], identification of when antibiotics are required will be insufficient to guide accurate treatment. With both viral and bacterial illnesses requiring targeted treatment, and the possibility of one or both being present, successful infection biomarkers must make a more nuanced diagnosis. Moreover, in childhood in particular, the incidence of bacterial infections has decreased considerably since the introduction of conjugate vaccines; it follows that the proportion of children presenting with febrile illness who have alternative diagnoses, including inflammatory conditions, is increasing. In addition to the decreasing burden of bacterial infection, the increased recognition of inflammatory illness in children may reflect changes in ascertainment, as well as true increases in incidence, as seen in the case of Kawasaki disease (12).

## Defining Bacterial Infections

Traditionally, most biomarker discovery studies have ascertained bacterial etiology based on bacterial detection by culture or PCR in a sterile site (including urine, CSF, blood; often referred to as “invasive bacterial infections”) (13–15). A patient without this evidence will then typically be classed as non-bacterial. Some studies, in particular those with a focus on pragmatic clinical translation, include positive cultures from non-sterile sites (e.g., throat swab, stools, skin) and imaging results such as radiographical changes on chest X-ray (e.g., to define bacterial pneumonia), CT or MRI (e.g., to define mastoiditis). Furthermore, intra-operative findings and histology (e.g., appendicitis, septic arthritis) or a clinical diagnosis without microbiological evidence (e.g., abscess, cellulitis) might be added to the outcome reference standard. This broader definition of complicated bacterial infections is often referred to as “serious bacterial infections” (Appendix A in Supplementary Material). In many studies, an expert opinion will be included to agree on the most appropriate final diagnosis. This has proven a fairly robust, but labor intensive approach to ensuring reproducibility between study outcomes (16, 17).

One of the major drawbacks of using bacterial cultures for confirming “definite bacterial” infection is their limited sensitivity (Table 1) (18). The sensitivity is directly related to the volume sampled, which is a well-known problem for blood cultures in neonates and children (19), to the prior use of antibiotics, which is very common in some settings (20), to culture techniques and to the types of pathogens. Other limitations of cultures of sterile sites are the high rates of contaminants, at times as high as the rate of true bacterial pathogens (21), and whether or not the site of infection can be sampled directly. Molecular strategies are now being employed to optimize the capture rate of pathogens in addition to conventional blood cultures. For instance, meningococcal PCR is already considered the gold standard confirmatory test (22, 23). In the UK-based multicenter DINOSAUR study molecular techniques improved the number of pathogens detected in children with convincing evidence of infective osteomyelitis or septic arthritis (24, 25). Molecular diagnostic panels, such as Septifast, Sepsitest, and Biofire Filmarray (26–30), have been shown to increase the number of positive findings in blood with relatively short turnaround time (31, 32). However, problems of sensitivity and specificity persist (33, 34), and studies that combine molecular and culture approaches still have disappointing diagnostic yield. For example, in the large observational study of children with life-threatening infection admitted to hospitals across several European countries (EUCLIDS), more than half of the children with a serious infection did not have a definitive causative pathogen identified, despite extensive diagnostic work-up (35). In addition, with bacterial identification, usually from non-sterile sites, the distinction between acute infection and carriage is often unclear, particularly in patients with co-morbidities.

**Table 1:** Challenges with the interpretation of biomarkers and diagnosing serious bacterial infection.

**[Difficulties in establishing diagnosis of serious bacterial infection]**
Material used for reference tests
Sensitivity of cultures and other molecular techniques/dependence on sampling volume
Material for testing not available in children (e.g., sputum)
Reduced culture sensitivity after antimicrobial treatment
Interpretation of test results
Pathogen isolated and the risk of being a contaminant
Pathogen causing infection vs. carriage vs. colonization
Multiple pathogens isolated
Isolated pathogen not in keeping with clinical phenotype
Applying the reference standard of SBI
Missing reference tests for cases suspected of SBI
A positive test result for a viral pathogen does not always exclude a bacterial infection
**[Influencing (the interpretation of) biomarker results]**
Age
Co-morbidities
Ethnicity
Use of immune- suppressive or modulatory drugs
Recent surgery, trauma, or other pro-inflammatory condition
Duration of symptoms
Vaccination status^a^
Traveling and exposure history
Primary care vs. secondary care and ED vs. PICU: differences in patient populations and the case-mix of settings differences in incidence of SBI differences in epidemiology: seasonality and endemic disease^a^
Cell cytopenia and limited protein, metabolite, or RNA yield

a *Differences in vaccination schemes and status, as well as seasonality and endemic disease can change the pre-test probabilities of the individual patient of having or not having (a specific type of) SBI, altering the interpretation of a biomarker result and its effect on the post-test probabilities and making a diagnosis of (a specific type of) SBI more or less likely; e.g., seasonality of enterovirus and influenzavirus can lead to different interpretation of biomarker value*.

*ED, emergency department; PICU, pediatric intensive care unit; SBI, serious bacterial infection*.

Defining bacterial pneumonia, the most common SBI with an overall mortality of 6.4 per 100,000 for children aged 5 years and under in high income countries (2, 3), is particularly challenging without a gold-standard diagnostic test. As collecting suitable diagnostic biosamples for the lower respiratory tract in children is difficult, a diagnosis will often be made based on chest X-ray changes or on clinical grounds alone, both of which are unreliable for establishing a definitive diagnosis of community acquired pneumonia (36, 37). Guidance by the World Health Organisation, albeit mostly applicable to lower income countries without referral capacity, recommends antibiotic treatment for community acquired pneumonia on fast breathing alone (38). Recent studies have improved our understanding of the etiology of childhood pneumonia using more elaborate diagnostic platforms. In the PERCH *(“Pneumonia Etiology Research for Child Health”*) study, conducted in several lower and middle income countries, viruses were the causative pathogen in the majority children with pneumonia, with RSV most commonly identified in approximately one third of children (39). However, in the absence of a sensitive diagnostic test, bacterial involvement cannot be ruled-out as contributory. A North American study on childhood pneumonia demonstrated that multiple viruses or bacteria or both viruses and bacteria were isolated in many children, in line with current thinking that respiratory tract infections are the result of complex mechanisms involving multiple organisms and varying host-immune responses (40, 41). Some viruses, in particular RSV and influenza virus, were more likely to be associated with disease than carriage (42–44).

## Role of Biomarkers

Many biomarkers have been proposed for differentiating viral and bacterial infections (45). The most evidence is available for CRP and PCT, and they appear equally useful in many clinical areas, even though neither can be used for diagnosing SBI with confidence in isolation (13, 46). PCT performs slightly better in young infants and neonates, and children with a short duration of fever compared with CRP (47), reflecting the differences in physiological inflammatory responses and time to elevated levels of CRP and PCT after stimulus (48). Despite the evidence available on its limited diagnostic utility, white cell count is still commonly used (13). Many other biomarkers have been explored, some with very promising initial results. For example, CD64 was useful in PICU settings, but did not validate well in ED settings (Table 1). Other markers of bacterial infection, such as neutrophil gelatinase-associated lipocalin-2 (NGAL) and resistin, have shown promise across a range of clinical settings but have not been integrated in clinical practice yet.

The pressure to improve early treatment of true bacterial infection, whilst avoiding unnecessary treatments, set against the decreasing incidence of bacterial illness and increasing incidence of inflammatory conditions makes the case for novel, accurate diagnostic strategies more compelling (49). Yet, despite many promising candidates (1, 50, 51), few biomarkers complete the journey from discovery to translation (52). An important obstacle in the development of bacterial biomarkers remains the lack of a consistent reference standard to classify SBIs, often aiming to capture a heterogeneous mix of causative pathogens and clinical phenotypes, not easily captured with a single, or minimal set of, biomarker(s) (35, 51, 53). Another obstacle to translation arises when biomarkers are discovered and perform well in high-incidence settings, for instance in severely unwell children in PICU with a clear or extreme presentation, but have poor performance in a low-incidence setting like emergency departments where they are most needed, where diagnostic uncertainty is higher, and clinical presentations less clear-cut. As different types of bacterial infections might need different diagnostic and management strategies, it seems unrealistic for biomarkers to be equally predictive for all. Some studies use a polytomous approach, allowing for different types of bacterial infections in their modeling (51), whilst other have looked at a single bacterial infection (54–58).

Future biomarker strategies, drawn from multi-omic discovery approaches, may enable classification of a wide range of febrile illnesses spanning bacterial and viral illness, other infections and inflammatory conditions, and also include other variables such as disease severity or prognosis. It is therefore essential that phenotyping approaches are able to classify the full range of presentations likely to be needed to be diagnosed in such a multi-class testing approach. With this paper we propose a novel classification framework to guide the design and evaluation of biomarker discovery and validation for childhood febrile illness, one which reflects the complex interplay between bacterial, viral and inflammatory illnesses. By means of illustrative validation studies using five prospective cohorts of children with infections, we aimed to evaluate the diagnostic performance of a new classification algorithm for biomarker discovery in children at risk of SBI.

## Methodology

Using five prospective, previously published, cohorts including children aged <16 years used for biomarker discovery and validation studies (Table 2) (59–62), we assessed the performance of the biomarkers procalcitonin (PCT, 4 cohorts), neutrophil gelatinase-associated lipocalin-2 (NGAL, 3 cohorts) and resistin (2 cohorts) to classify patients as having “bacterial” infection. These biomarkers were measured in each of the local reference laboratories, as detailed in the original publications.

**Table 2:** Description of cohorts.

	**Setting**	**Participants**	**Exclusion**	**Design**	**Biomarkers**	**Incidence**	**Study characteristics**
Alder Hey ED cohort (59)	Pediatric emergency department, tertiary hospital, UK	Febrile children aged <16 years attending the ED in whom blood test were being performed, 2010-2012, *n* = 1,183	Primary immunodeficiency	Prospective observational	PCT (*n* = 1,107) Resistin (*n* = 1,119) NGAL (*n* = 1,120)	SBI: 338 (29%)^a^Definite bacterial: 82 (7%)Definite viral: 94 (8%)	Median age: 2.5 years (IQR 0.9–5.7 years); 654 (55%) were boys. 1/3 of children had comorbidities.
Alder Hey PICU cohort (60)	PICU, tertiary hospital, UK	Consecutive children aged <16 years admitted to PICU with suspected infection or developed infection after admission, 2010–2012, *n* = 352	For this study: Final diagnosis: no concerns for infection Only biomarkers on day infection suspected were considered for this study	Prospective observational	PCT (*n* = 346) Resistin (*n* = 184) NGAL (*n* = 182)	SBI: 83 (24%)^a^Definite bacterial: 48 (14%)Definite viral: 48 (14%)	Median age: 1.3 years (IQR 0.4–5.4 years); 203 (58%) were boys.
**Erasmus ED cohort** (61)	Pediatric emergency department, tertiary hospital, NL	Consecutive children aged 1 month−16 years with fever, 2009–2012, *n* = 1,993	Chronic underlying disease; well appearing children presenting with fever and a clear focus of an upper airway infection; revisit to ED within 5 days	Prospective observational	PCT (*n* = 710)	SBI: 230 (12%)^a^Definite bacterial: 71 (4%)Definite viral: 109 (5%)	Median age 1.8 (IQR 0.9–3.9); 1,094 (55% were boys)
**Maasstad ED Cohort** **(** **61** **)**	Pediatric emergency department, district general hospital, NL	Consecutive children aged 1 month−16 years with fever, 2011–2012, *n* = 714	Chronic underlying disease; well appearing children presenting with fever and a clear focus of an upper airway infection; revisit to ED within 5 days	Prospective observational	PCT (*n* = 386)	SBI: 103 (14%)^a^Definite bacterial: 46 (6%)Definite viral: 52 (7%)	Median age 1.6 (IQR 0.7–3.6); 399 (56%) were boys
**St. Mary's hospital cohort** **(** **62** **)**	Pediatric clinical areas, tertiary hospital, UK	Acutely ill febrile children, aged <17 years, with illness of sufficient severity warranting blood tests, 2009–2012, *n* = 394 including 67 controls	Sample of *n* = 200 selected for this biomarker study, including *n* = 40 controls	Prospective observational	NGAL (*n* = 200)	SBI: 87 (54%)^c^Definite bacterial: 38 (24%)^bc^Definite viral: 43 (27%)^c^	Median age 3.3 (IQR 1.8–7.1); 108 (54%) were boys

a *Outcome of interest in original study: SBI vs. non-SBI*.

b *Outcome of interest in original study: Definite bacterial vs. probable bacterial vs. unknown vs. definite viral vs. other vs. control*.

c *Out of 160 patients (excluding controls)*.

*ED, emergency department; IQR, interquartile range; NGAL, neutrophil gelatinase-associated lipocalin; PCT, procalcitonin; PICU, pediatric intensive care unit; SBI, serious bacterial infection*.

The Alder Hey ED (*n* = 1,183), Maasstad ED (*n* = 714) and Erasmus ED (*n* = 1,993) cohorts recruited consecutive febrile children presenting to the emergency department in whom additional blood tests were done; the research biosamples for the ED cohorts were taken as additional samples at the time of taking the initial blood tests, and ideally before the administration of systemic antibiotics. The Alder Hey PICU cohort included children with suspected infection in the pediatric intensive care unit (*n* = 352), with research biosamples taken on admission to PICU or at the time of developing an infection during PICU stay; the St. Mary's hospital cohort recruited acutely ill febrile children admitted to pediatric wards or intensive care (*n* = 394), and research biosamples were taken at the earliest opportunity during the patient's inpatient stay.

**Figure 2:**
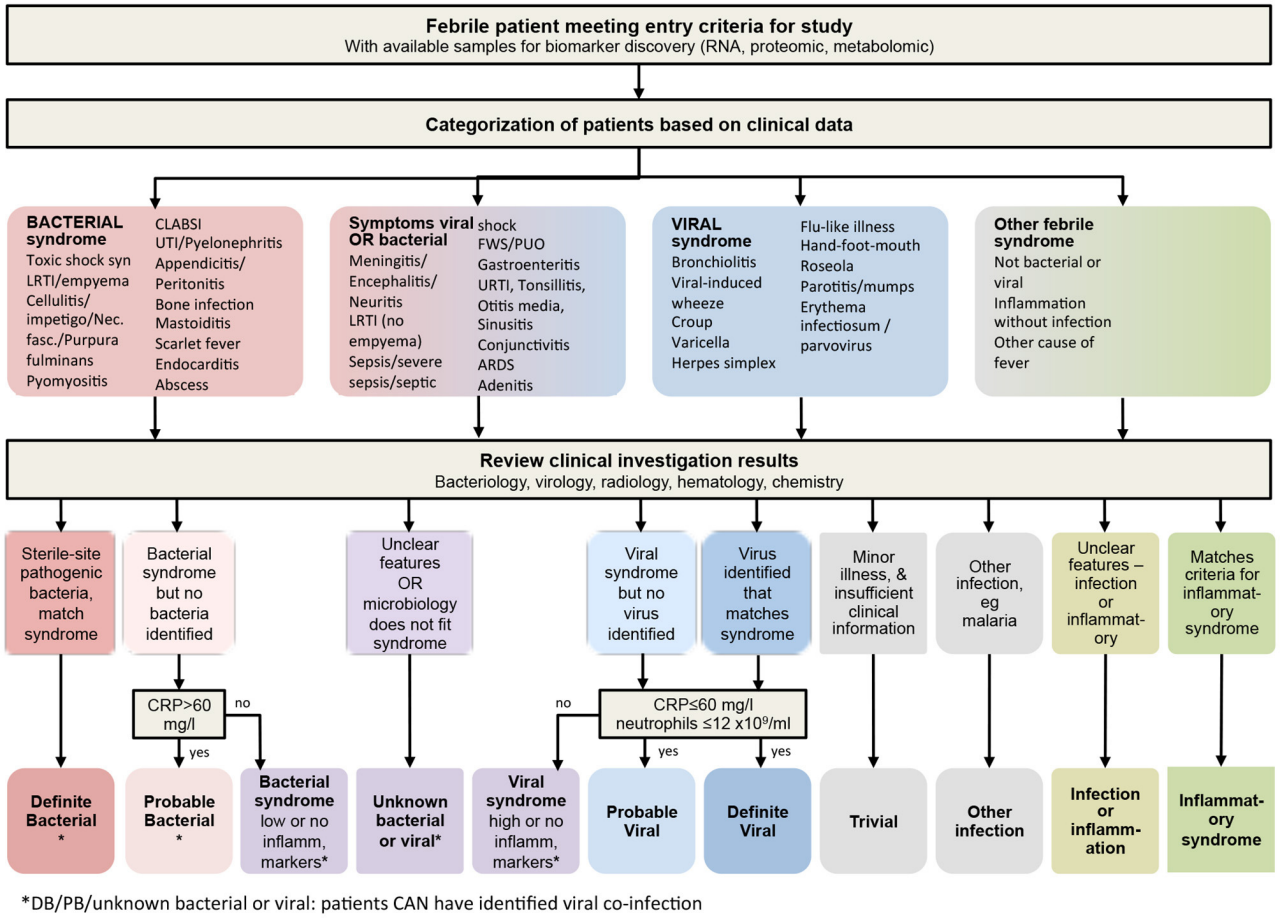
Algorithm for classifying children at risk of serious infection. Following discharge, clinical phenotypes were assigned after review of all available clinical and laboratory data including biochemistry, hematology, radiology and microbiology. Children allocated to the “other infection,” “infection or inflammation,” or “inflammatory syndrome” boxes at the bottom right would normally be analyzed as its component parts individually, so that studies can recruit and meaningfully analyze data from these type of patients alongside the infection patients. CRP, C-reactive protein.

We then re-categorized the children of these four cohorts, blinded for our biomarkers of interest, from the original dichotomous SBI classification into one of the eleven distinct outcome groups in view of their likelihood of having a bacterial or viral infection, or both (Figure 2), and using an extended version of a published algorithm previously used to derive a 2-transcript bacterial-viral diagnostic classifier (63). For the fifth cohort, i.e., the St. Mary's hospital cohort, we allocated final diagnoses for both the SBI and the PERFORM classification systems, blinded for the biomarkers of interest. The PERFORM algorithm broadly groups patients into patients with a likely bacterial infection, patients with a likely viral infection, patients with unknown viral and/or bacterial infection and other febrile syndromes, which includes patients with suspected or confirmed inflammatory conditions, and infections with distinct treatments or non-viral/bacterial etiology, such as tuberculosis and malaria (Figure 2). We then examined the distribution of the biomarkers according to the patient classifications. For this study, we combined “trivial,” “other infection,” “infection or inflammation” and “inflammatory syndrome” into one “Other” group; with the cohorts having few or no cases.

Concentrations of biomarkers were visualized using barplots with median concentration levels and interquartile ranges, and these were compared for “SBI” vs. “non-SBI” (the SBI algorithm, Appendix A in Supplementary Material) and “definite bacterial” vs. “definite viral” (i.e., the groups of the PERFORM algorithm with most diagnostic certainty, Figure 2) using Wilcoxon non-parametric rank sum tests, and for all levels of the PERFORM algorithm using Kruskal-Wallis tests. Spearman correlation coefficients were calculated for the concentrations of biomarkers and categories with viral and bacterial infections of the PERFORM algorithm. Pearson correlation coefficients were calculated for the correlation between C-Reactive Protein (CRP), which was used to allocate a final diagnosis in the PERFORM algorithm and the biomarkers PCR, NGAL and resistin. We compared the “SBI” and “PERFORM” phenotyping classification systems by measuring the Area Under the receiver operating Curves (AUC) of the biomarkers' ability to discriminate the predicted bacterial and viral groups, by means of “SBI” vs. “non-SBI” and “definite bacterial” vs. “definite viral” infection. Additionally, we calculated the AUC for a model that combined PCT, NGAL and resistin using the data from the Alder Hey ED and Alder Hey PICU cohort, applying restricted cubic splines for optimal model fit. Only cases with available biomarker data were used. We calculated summary AUCs using random effect models and presented these in forest plots. All analyses were performed in R v4.0.0, including the use of packages pROC, ggpubr, and metafor.

## Results

The number of children diagnosed with SBI varied between the 5 cohorts, ranging from 12% in the Erasmus ED cohort to 54% in the St. Mary's hospital cohort (Table 2). For children with a definite bacterial (DB) infection this ranged from 4% in the Erasmus ED, to 24% in the St. Mary's hospital cohort, and for children with a definite viral infection (DV) this ranged between 5% (Erasmus ED cohort) and 27% (St. Mary's hospital cohort). Pneumonia was the most commonly diagnosed SBI in the Erasmus ED cohort (*n* = 107, 5%), the Alder Hey ED cohort (*n* = 107, 9%), and the St. Mary's hospital cohort (*n* = 57, 29%); in the Maastad ED cohort this was urinary tract infection (*n* = 42, 6%) and in the Alder Hey PICU cohort it was sepsis (*n* = 62, 18%; of which 11 (3%) were culture-negative sepsis). Biomarker levels were markedly higher in all types of infection in the Alder Hey PICU cohort and St. Mary's hospital cohort compared with the ED cohorts (Table 3). For both the SBI algorithm and the PERFORM algorithm, children with presumed bacterial infections had higher concentrations of PCT, NGAL and resistin than children with presumed viral infections or “non-SBI” (Table 3). Significant Spearman **ρ** coefficients (range −0.10 to −0.53 across biomarkers and cohorts) were observed for concentrations of PCT, NGAL and resistin and the full spectrum of diagnostic groups of the PERFORM algorithm, with higher concentrations for children with bacterial infection compared to those with viral infections (Table 3). The biomarkers NGAL (Pearson correlation coefficient: range 0.23–0.40), PCT (range: 0.25–0.41) and resistin (range: 0.14–0.21) had moderate correlation with CRP (Appendix B in Supplementary Material).

**Table 3:** Concentrations of biomarkers.

		**SBI classification**		**PERFORM classification**							
		**SBI**	**Non-SBI**	**Definite bacterial**	**Probable bacterial**	**Bacterial Syndrome**	**Unknown**	**Viral syndrome**	**Probable viral**	**Definite viral**	**Spearman ρ**
**Alder Hey ED cohort**, ***n*****=1,161**											
NGAL (ng/mL)	78.1	102.5	70.2	140.0	105.9	83.6	77.2	99.0	63.4	75.4	−0.18^*^
	(52.5–121.1)	(66.2–159.5)	(49.6–102.2)	(82.1 −228.8)	(80.7–160.0)	(57.9–123.4)	(63.1–101.8)	(68.1–148.8)	(43.8–93.8)	(53.3–101.9)	
	*N* = 1,120	*N* = 322	*N* = 798	*N* = 78	*N* = 169	*N* = 107	*N* = 33	*N* = 46	*N* = 559	*N* = 88	
Resistin (ng/L)	40.3	59.4	35.7	67.8	59.8	45.1	27.0	40.6	33.9	35.6	−0.10^*^
	(21.5–73.7)	(29.7–104.4)	(19.3–63.8)	(34.8–124.1)	(33.6–105.0)	(28.1–87.7)	(16.7–52.9)	(18.8–73.7)	(18.6–61.9)	(17.6–63.0)	
	*N* = 1,119	*N* = 321	*N* = 798	*N* = 78	*N* = 168	*N* = 107	*N* = 33	*N* = 46	*N* = 559	*N* = 88	
PCT (μg/L)	0.23	0.47	0.18	2.40	1.08	0.16	0.20	0.65	0.15	0.21	−0.18^*^
	(0.10–0.80)	(0.13–3.13)	(0.09–0.53)	(0.26–10.95)	(0.28–3.00)	(0.09–0.52)	(0.10–0.52)	(0.33–1.40)	(0.08–0.38)	(0.13–0.54)	
	*N* = 1,107	*N* = 320	*N* = 787	*N* = 79	*N* = 167	*N* = 106	*N* = 31	*N* = 46	*N* = 551	*N* = 86	
**Alder Hey PICU cohort**, ***n*****=352**^**∧**^											
NGAL (ng/mL)	116.0	167.1	110.3	170.1	126.1	102.8	125.3	93.2	–	72.1	−0.27^*^
	(70.5–198.5)	(82.4–302.1)	(68.5–175·6)	(91.7–291.5)	(97.2–229.1)	(71.8–189.9)	(71.2–227.1)	(42.7–157.0)		(51.3–116.7)	
	*N* = 182	*N* = 42	*N* = 142	*N* = 25	*N* = 34	*N* = 36	*N* = 49	*N* = 4	*N =* 1	*N* = 48	
Resistin (ng/L)	54.4	58.2	50.3	57.1	73.8	45.6	46.3	80.7	–	37.3	−0.22^*^
	(29.1–97.8)	(31.8–158.9)	(28.2–87.6)	(26.1–169.3)	(48.5–155.4)	(31.1–87.4)	(31.6–73.6)	(50.5–110.2)		(13.4–67.1)	
	*N* = 184	*N* = 43	*N* = 141	*N* = 28	*N* = 35	*N* = 38	*N* = 46	*N* = 4	*N* = 0	*N* = 33	
PCT (μg/L)	0.64	4.40	0·41	12.95	1.00	0.28	0.43	8.2	–	0.38	−0.23^*^
	(0.10–5.58)	(0.29–49.05)	(0.09–2.33)	(1.07–98.0)	(0.18–8.30)	(0.08–2.41)	(0.09–2.55)	(8.1–23.3)		(0.11–0.83)	
	*N* = 346	*N* = 82	N = 264	*N* = 48	*N* = 73	*N* = 79	*N* = 92	*N* = 5	*N* = 1	*N* = 48	
**Erasmus ED cohort**, ***n*****=710**											
PCT (μg/L)	0.18	0.64	0.16	0.64	1.49	0.23	0.14	0.52	0.14	0.15	−0.34^*^
	(0.10–0.54)	(0.24–3.46)	(0.09–0.40)	(0.24–3.02)	(0.54–6.19)	(0.11–0.67)	(0.08–0.24)	(0.21–1.92)	(0.09–0.29)	(0.09–0.35)	
	*N* = 710	*N* = 103	*N* = 607	*N* = 34	*N* = 52	*N* = 61	*N* = 25	*N* = 43	*N* = 442	*N* = 53	
**Maasstad ED cohort**, ***n*****=386**											
PCT (μg/L)	0.21	1.03	0.17	1.36	1.76	0.17	0.10	0.43	0.16	0.18	−0.27^*^
	(0.10–0.67)	(0.28–2.61)	(0.09–0.42)	(0.33–2.38)	(0.35–4.96)	(0.07–0.42)	(0.05–0.30)	(0.21–1.53)	(0.09 0.35)	(0.10–0.60)	
	*N* = 386	*N* = 68	*N* = 318	*N =* 30	*N* = 30	*N =* 35	*N* = 14	*N* = 28	*N* = 215	*N* = 34	
**St. Mary's hospital cohort**, ***n*****=200**^**∧**^											
NGAL (ng/mL)	132.1	234.8	93.7	285.2	187.3	–	144.0	–	–	97.9	−0.53^*^
	(76.6–248.9)	(142.4–391.2)	(62.1–144.9)	(235.3–639.8)	(118.4–308.3)		(89.7–209.3)			(62.7–161.2)	
	*N* = 200	*N =* 87	*N* = 113	*N* = 38	*N* = 38	n/a	*N =* 32	n/a	n/a	*N* = 43	

*All concentrations in median and interquartile range (IQR); median (IQR) not shown for the “Other” category. ^∧^The Alder Hey PICU cohort only had minimal cases of “probable viral” or “viral syndrome”; The St. Mary's hospital cohort had no cases coded as “bacterial syndrome,” “viral syndrome” or “probable viral.”*

* *Spearman ρ p-value < 0.05 for correlation of the full spectrum of the PERFORM classification*.

*ED, emergency department; NGAL, neutrophil gelatinase-associated lipocalin; PCT, procalcitonin; PICU, pediatric intensive care unit; SBI, serious bacterial infection*.

**Figure 3:**
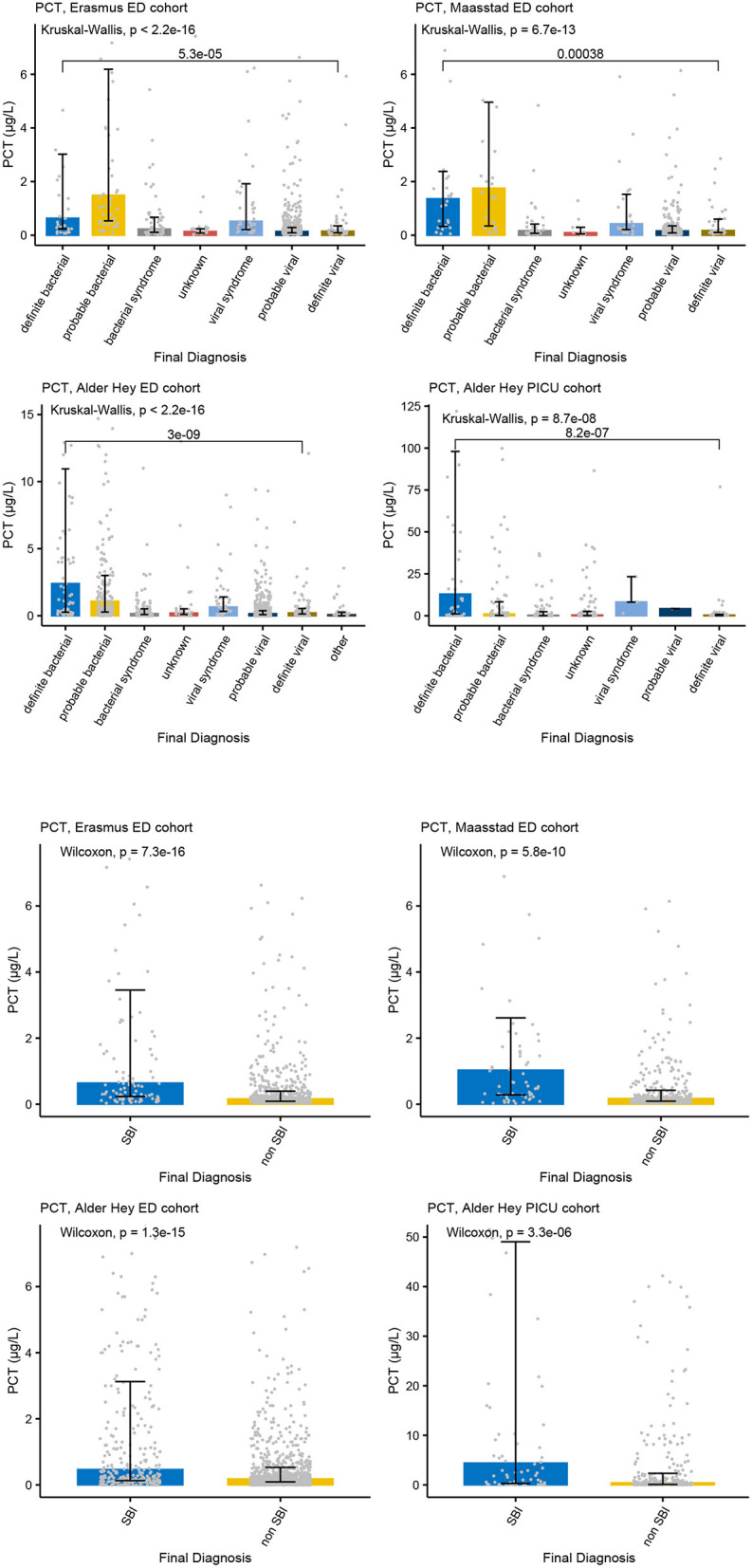
PCT and serious bacterial infections. Each graph shows the concentrations of PCT (microgr/L) for each of the categories of the PERFORM classification algorithm (top two rows) and of the SBI classification algorithm (bottom two rows) in the Erasmus cohort (left, 1st and 3nd row), Maasstad cohort (right, 1st and 3rd row), Alder Hey ED cohort (left, 2nd and 4th row), and the Alder Hey PICU cohort (right, 2nd and 4th row). Each bar represents median concentration values, with the black lines representing the interquartile range, and the gray dots representing individual values. Overall significance for the PERFORM classification algorithm is given using the Kruskal Wallis test, and for the SBI classification using the Wilcoxon rank sum test. In addition, significance value for “definite bacterial” vs. “definite viral” of the PERFORM algorithm was calculated using the Wilcoxon rank sum test.

**Figure 4:**
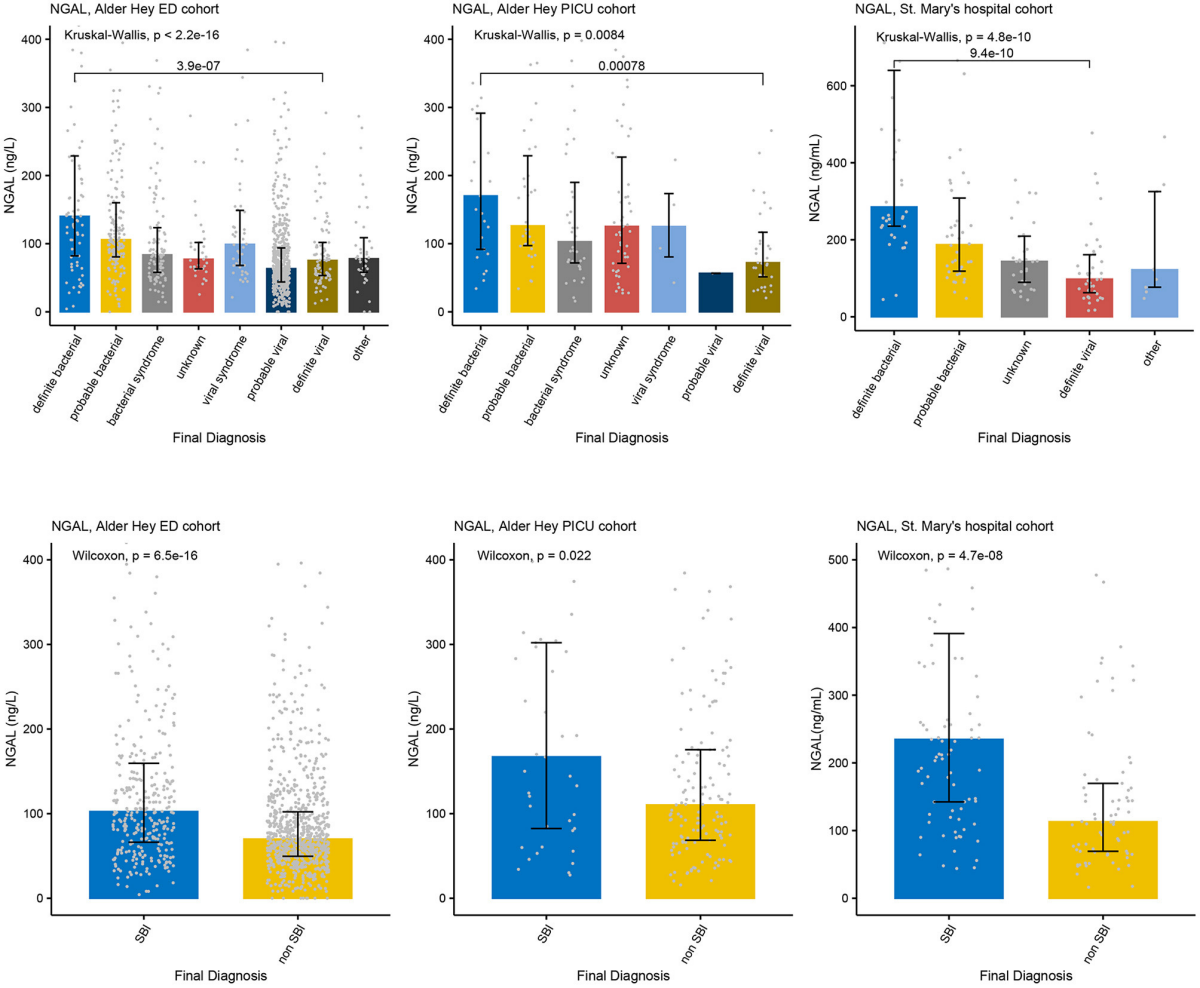
Concentrations of NGAL. Each graph shows the concentrations of NGAL (ng/L) for each of the categories of the PERFORM classification algorithm (top row) and of the SBI classification algorithm (bottom row) in the Alder Hey ED cohort (left), the Alder Hey PICU cohort (middle) and the St. Mary's hospital cohort (right). Each bar represents median concentration values, with the black lines representing the interquartile range, and the gray dots representing individual values. Overall significance for the PERFORM classification algorithm is given using the Kruskal Wallis test, and for the SBI classification using the Wilcoxon rank sum test. In addition, significance value for “definite bacterial” vs. “definite viral” of the PERFORM algorithm was calculated using the Wilcoxon rank sum test.

**Figure 5:**
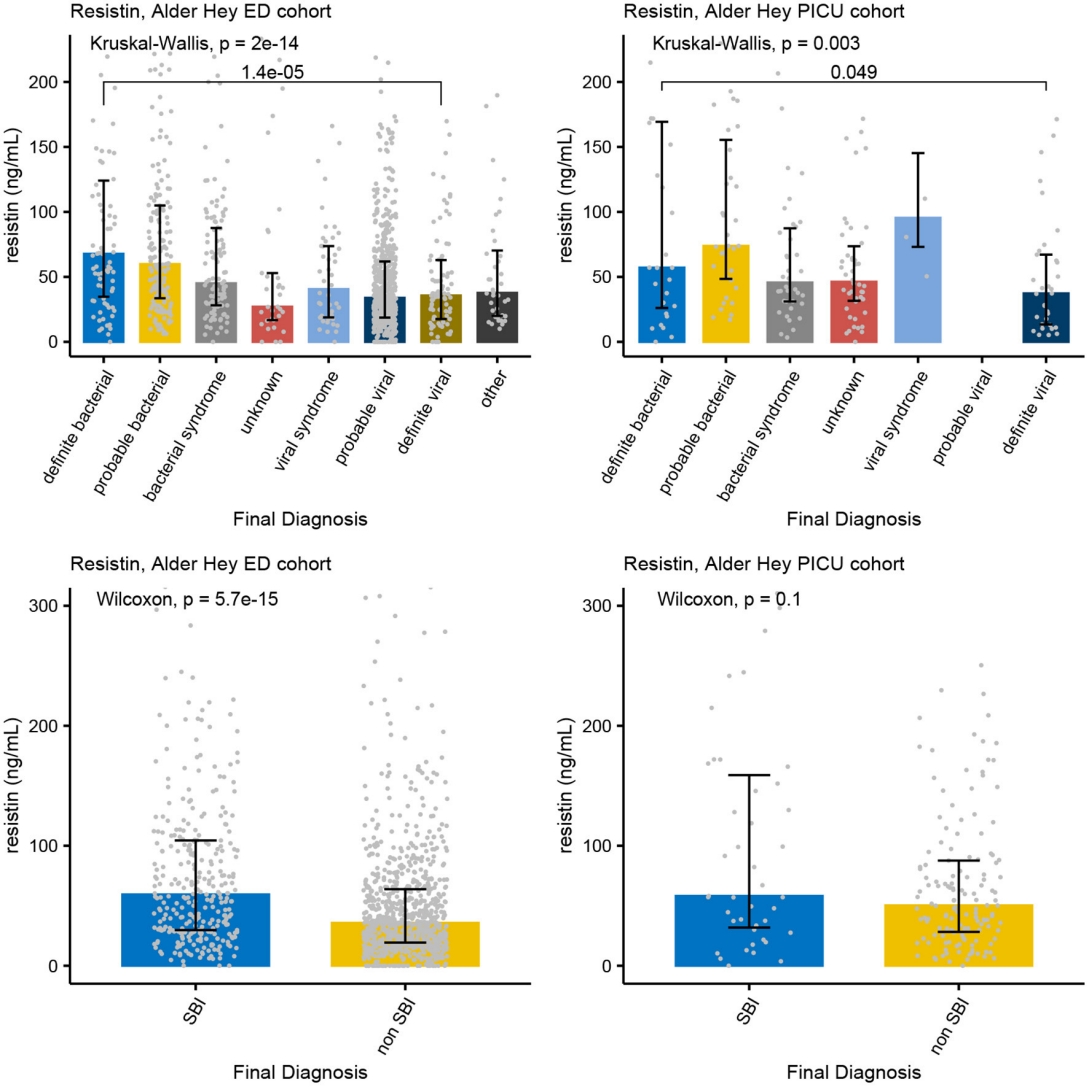
Concentrations of Resistin. Each graph shows the concentrations of Resistin (ng/L) for each of the categories of the PERFORM classification algorithm (top row) and of the SBI classification algorithm (bottom row) in the Alder Hey ED cohort (left) and the Alder Hey PICU cohort (right). Each bar represents median concentration values, with the black lines representing the interquartile range, and the gray dots representing individual values. Overall significance for the PERFORM classification algorithm is given using the Kruskal Wallis test, and for the SBI classification using the Wilcoxon rank sum test. In addition, significance value for “definite bacterial” vs. “definite viral” of the PERFORM algorithm was calculated using the Wilcoxon rank sum test.

Increased concentrations of PCT were strongly associated with bacterial infections in all four cohorts for both the PERFORM and SBI algorithms, and the PERFORM classification algorithm showed a clear trend toward higher concentrations of PCT in children with a higher degree of certainty of having a bacterial infection (Figure 3). NGAL concentrations differed between bacterial and viral infections as per PERFORM algorithm, as well as between “SBI” and “non-SBI,” in the Alder Hey ED, the Alder Hey PICU and the St Mary's hospital cohorts (Figure 4). Resistin levels did not discriminate “SBI” from “non-SBI” in the Alder Hey PICU cohort (Figure 5). Notably, children in “viral syndrome” or “unknown” groups of the PERFORM algorithm had high levels of PCT and NGAL.

**Figure 6:**
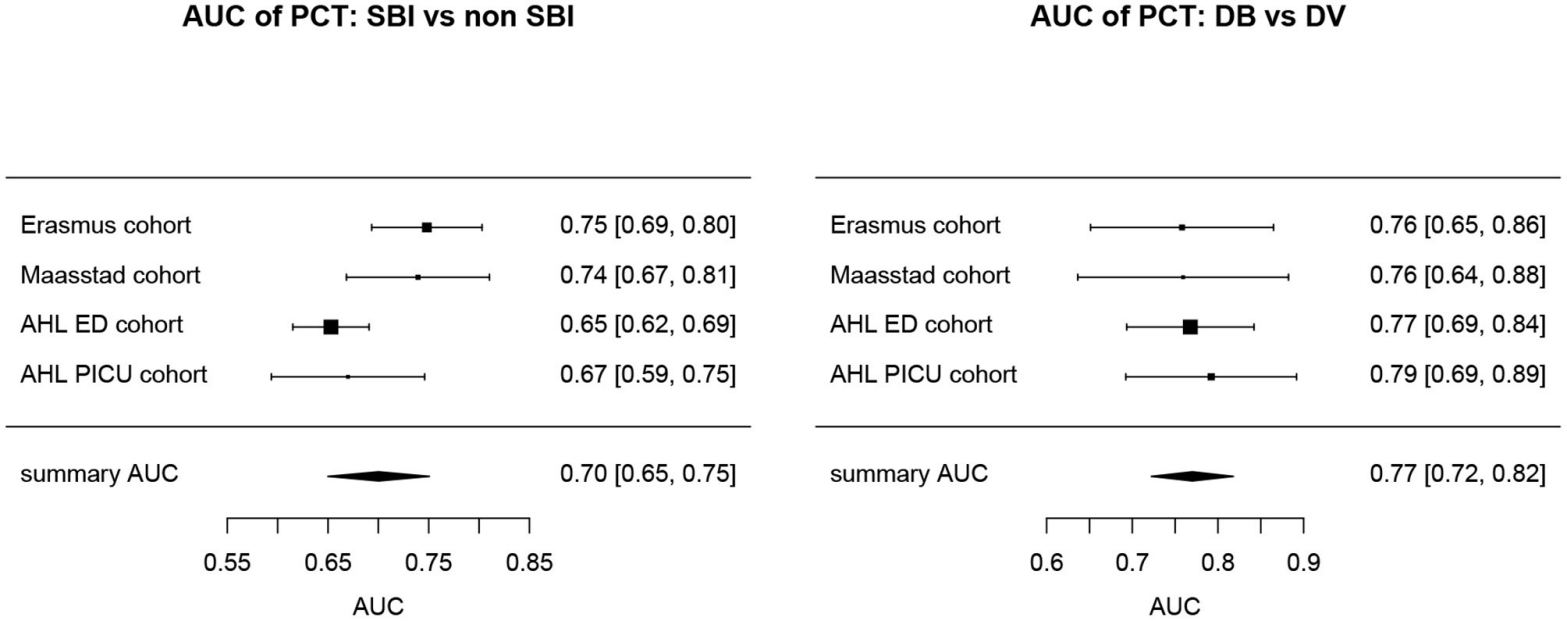
Forest plot of summary AUC: PCT. Forest plot of random effects model of the AUCs of PCT predicting SBI vs. non-SBI (left) and Definite Bacterial (DB) vs. Definite Viral (DV) (right) for our four cohorts with PCT available (y-axis). The black squares show the mean AUC values with the 95% confidence intervals on the x-axis. Overall summary AUC and confidence interval are shows as black diamond. For SBI vs. non-SBI: model *I*^2^ = 67.26%, test for heterogeneity Q (df = 3) = 101,349, *p*-value 0.0175; for DB vs. DV: model *I*^2^ = 0.00%, test for heterogeneity Q (df = 3) = 0.2694, *p*-value 0.9657.

**Figure 7:**
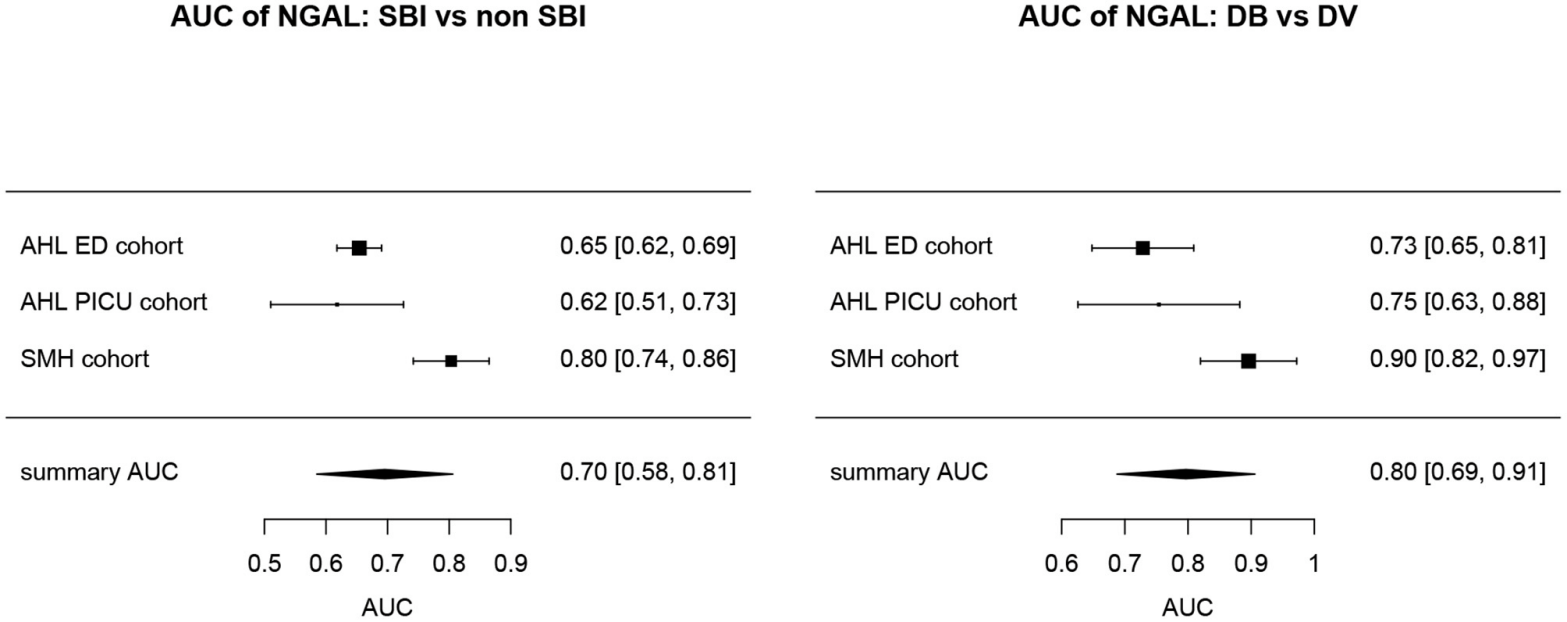
Forest plot of summary AUC: NGAL. Forest plot of random effects model of the AUCs of NGAL predicting SBI vs. non-SBI (left) and Definite Bacterial (DB) vs. Definite Viral (DV) (right) for our three cohorts with NGAL available (y-axis). The black squares show the mean AUC values with the 95% confidence intervals on the x-axis. Overall summary AUC and confidence interval are shows as black diamond. For SBI vs. non-SBI: model *I*^2^ = 89.14%, test for heterogeneity Q (df = 2) = 18.4711, *p*-value < 0.001; for DB vs. DV: model *I*^2^ = 76.23%, test for heterogeneity Q (df = 2) = 9.5315, *p*-value 0.0085.

**Figure 8:**
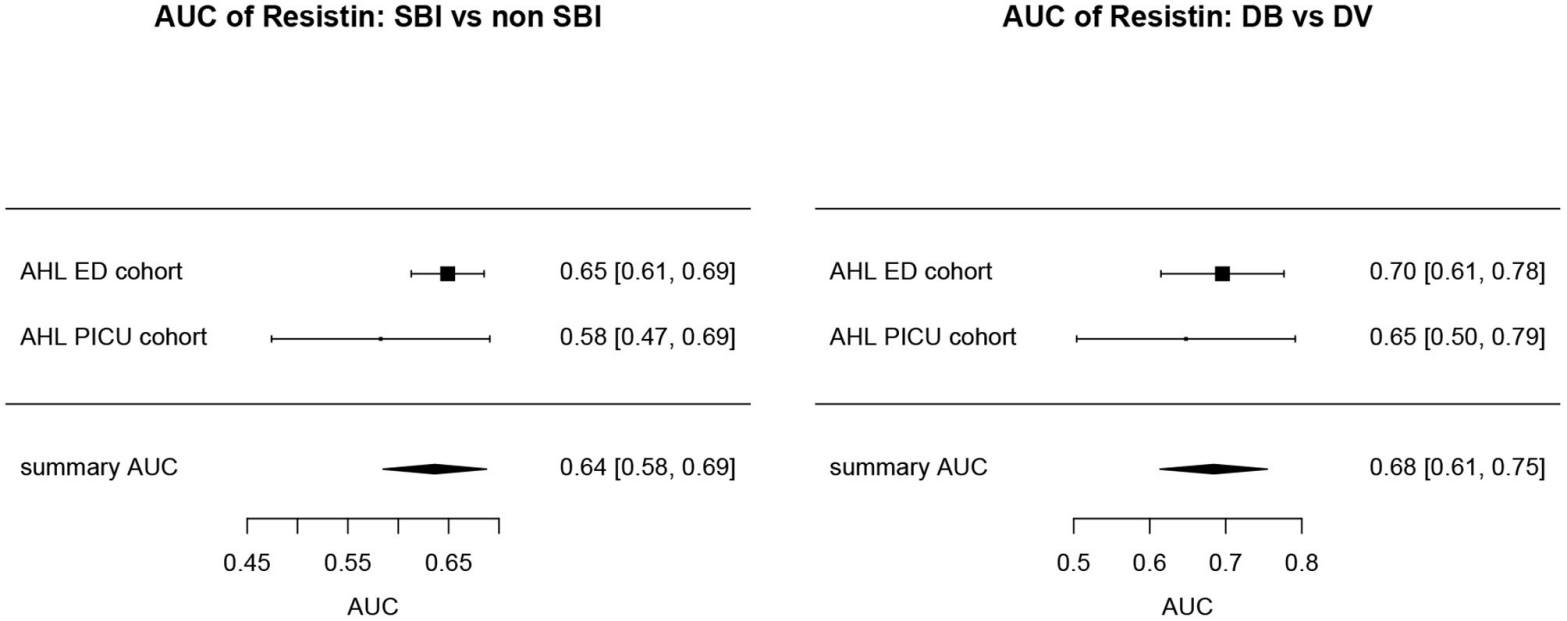
Forest plot of summary AUC: Resistin. Forest plot of random effects model of the AUCs of Resistin predicting SBI vs. non-SBI (left) and Definite Bacterial (DB) vs. Definite Viral (DV) (right) for our two cohorts with Resisting available (y-axis). The black squares show the mean AUC values with the 95% confidence intervals on the x-axis. Overall summary AUC and confidence interval are shows as black diamond. For SBI vs. non-SBI: model *I*^2^ = 23.13%, test for heterogeneity Q (df = 1) = 1.3009, *p*-value 0.2540; for DB vs. DV: model *I*^2^ = 0.00%, test for heterogeneity Q (df = 1) = 0.3248, *p*-value 0.5687.

**Figure 9:**
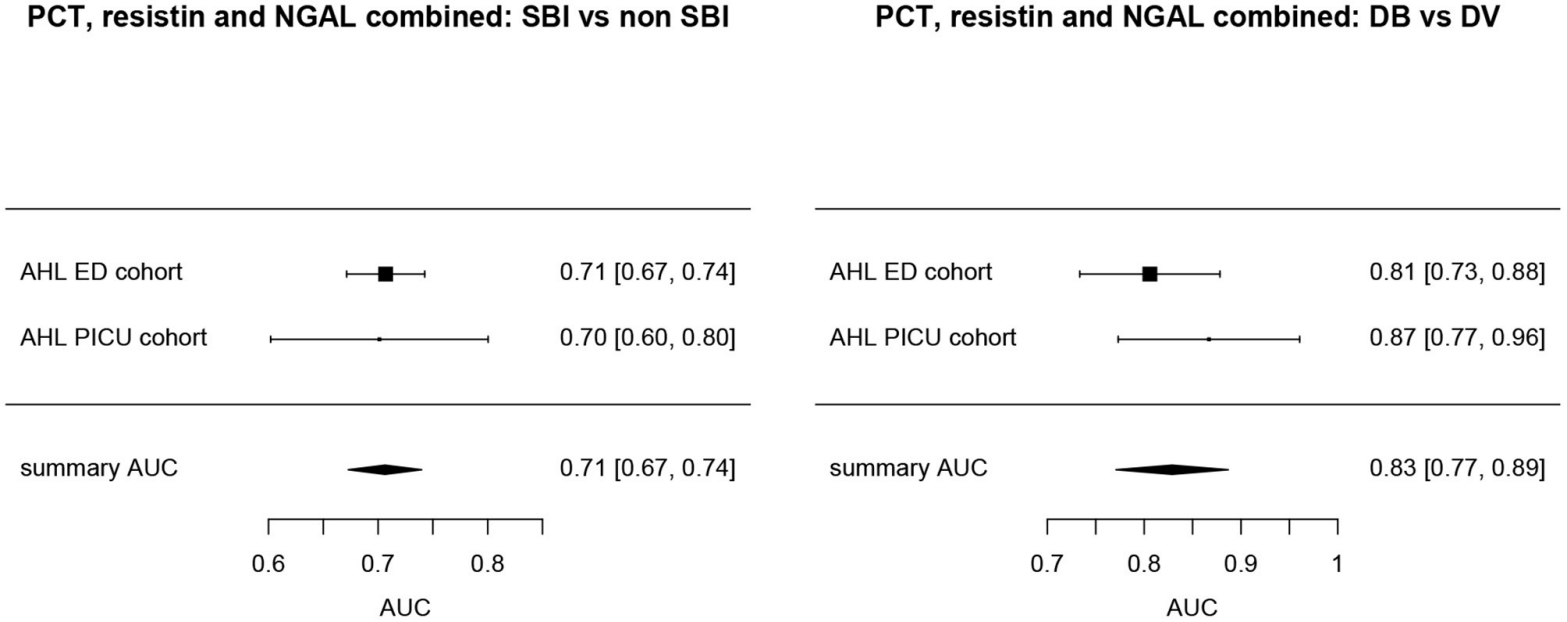
Forest plot of summary AUC: PCT, Resistin and NGAL combined. Forest plot of random effects model of the AUCs of PCT, Resistin and NGAL combined predicting SBI vs. non-SBI (left) and Definite Bacterial (DB) vs. Definite Viral (DV) (right) for our two cohorts with all three biomarkers available (y-axis). The black squares show the mean AUC values with the 95% confidence intervals on the x-axis. Overall summary AUC and confidence interval are shows as black diamond. For SBI vs. non-SBI: model *I*^2^ = 0.00%, test for heterogeneity Q (df = 1) = 0.0112, *p*-value 0.9156; for DB vs. DV: model *I*^2^ = 2.02%, test for heterogeneity Q (df = 1) = 1.0206, *p*-value 0.3124.

Overall, AUCs for “definite bacterial” infections vs. “definite viral” infections were higher than AUCs for “SBI” vs “non-SBI” in all cohorts (Table 4). PCT gave a summary AUC of 0.77 (95% CI 0.72–0.82) for discriminating “definite bacterial” (DB) infections (*n* = 191) from “definite viral” (DV) infections (*n* = 222) according to the PERFORM algorithm vs. 0.70 (95% CI 0.65–0.75) for discriminating “SBI” (*n* = 573) from “non-SBI” (*n* = 1,976) (Figure 6); for NGAL this was 0.80 (95% CI 0.69–0.91; with DB, *n* = 141; DV, *n* = 179) vs. 0.70 (95% CI 0.58–0.81; with SBI, *n* = 451; non-SBI, *n* = 1,053) (Figure 7); for resistin this was 0.68 (95% CI 0.61–0.75; with DB, *n* = 106; DV, *n* = 121) vs. 0.64 (95% CI 0.58–0.69; with SBI, *n* = 364; non-SBI, *n* = 939) (Figure 8). Combining PCT, resistin and NGAL in the Alder Hey ED and Alder Hey PICU cohorts improved the summary AUC more substantially for models predicting “definite bacterial” infections vs. “definite viral” infections [summary AUC of 0.83 (95% CI 0.77–0.89)] compared with “SBI” vs. “non-SBI” [summary AUC of 0.71 (95% CI 0.67–0.74)] (Figure 9).

**Table 4:** Diagnostic performance of biomarkers.

	**AUC**: **SBI vs. non-SBI** **(95% CI)**	**AUC:** **DB vs. DV** **(95% CI)**
**Alder Hey ED cohort**		
NGAL (ng/mL)	0.65 (0.62–0.69)	0.73 (0.65–0.81)
Resistin (ng/mL)	0.65 (0.61–0.69)	0.70 (0.61–0.78)
PCT (μg/L)	0.65 (0.62–0.69)	0.79 (0.69–0.89)
NGAL, resistin, PCT	0.71 (0.67–0.84)	0.81 (0.73–0.88)
**Alder Hey PICU cohort**		
NGAL (ng/mL)	0.62 (0.51–0.73)	0.75 (0.63–0.88)
Resistin (ng/mL)	0.58 (0.47–0.69)	0.65 (0.50–0.79)
PCT (μg/L)	0.67 (0.59–0.75)	0.77 (0.69–0.84)
NGAL, resistin, PCT	0.70 (0.60–0.80)	0.87 (0.77–0.96)
**Erasmus ED cohort**		
PCT (μg/L)	0.75 (0.69–0.80)	0.76 (0.65–0.86)
**Maasstad ED cohort**		
PCT (μg/L)	0.74 (0.67–0.81)	0.76 (0.64–0.88)
**St. Mary's hospital cohort**		
NGAL (ng/mL)	0.80 (0.74–0.86)	0.90 (0.82–0.97)

*DB, definite bacterial; DV, definite viral; NGAL, neutrophil gelatinase-associated lipocalin; PCT, procalcitonin; SBI, serious bacterial infection; AUC, area under the receiver operating curve; CI, confidence interval*.

## Discussions

Compared to the dichotomous categories from the original publications (“SBI” vs. “non-SBI”), the new PERFORM algorithm showed better discrimination and granularity across the full spectrum from “definite bacterial” to “definite viral.” It aligned well with host response biomarker concentrations, which had highest concentrations in the group with most certainty. Hence, the PERFORM algorithm helped define those with a bacterial infection, as well as those without a bacterial illness. This was seen across a range of clinical settings with varying incidences of bacterial infections, reflecting different recruitment strategies and supporting the broad applicability of the PERFORM algorithm. A combination of PCT, NGAL and resistin improved discrimination compared with the individual biomarkers in the Alder Hey ED and PICU cohorts, and more so for differentiating between “definite bacterial” infections and “definite viral” infections based on the PERFORM algorithm than for “SBI” and “non-SBI.” In the PERFORM algorithm, children with a clinical phenotype resembling a viral infection, but with a high CRP level not clearly explained by the presence of a bacterial co-infection, will be classified in either the “viral syndrome” or “unknown” group. Hence, high concentrations of PCT and NGAL in these two groups might represent misclassification or co-infection and are of interest for future biomarker studies. Even though there was only moderate correlation between the biomarkers of interest and CRP, using CRP to guide the phenotyping in the PERFORM algorithm might have increased the diagnostic performance of PCT, NGAL, and resisitin.

When we examined biomarker concentrations in children with unclear etiology for their illness (children with no positive microbiology, or in whom the microbiology does not fit the diagnostic phenotype) and all viral infections (“probable” and “definite”), there was a trend to a stepwise decrease in the median biomarker values, moving from most to least likely bacterial infection. Within each phenotypic category we found a range of biomarker concentrations spanning from “bacterial” level to “viral” range, as well as some showing intermediate values. The PERFORM phenotyping approach enabled categorization of children with more granularity. The data are consistent with the emerging evidence for a complex relationship between bacterial and viral pathogens in the etiology of disease, such that the overall clinical presentation may be a result of interplay between pathogens, including between bacteria and viruses (41). The PERFORM algorithm will also allow for the accurate classification of children with inflammatory conditions and other type of infections. Although there were few of these cases in our cohorts, it will be important to optimize their phenotyping, as illustrated by the emergence of the SARS-CoV-2 associated Multisystem Inflammatory Syndrome in children (MIS-C) (64).

The PERFORM classification algorithm aptly captures the degree of uncertainty of the final diagnosis, increasing the likelihood of a successful candidate biomarker to perform well in validation cohort studies. Furthermore, the algorithm gives insights in the distribution of the types of infection in different clinical settings. We now suggest, as a next step toward clinical implementation, to select the most promising candidate biomarkers with the most convincing trend of biomarker concentrations, and the best discriminative ability for “definite bacterial” vs. “definite viral” infection. Including those children with “probable bacterial” or “probable viral” infections can be considered to cover a wider range of clinical phenotypes. Then, candidate biomarkers should be validated in independent validation cohorts. Validation studies should recruit cohorts with consecutive patients, including those with an unclear clinical phenotype, and should be conducted in various clinical settings including low and middle income countries, as well as high and low incidence settings. To illustrate the importance of this, we showed that biomarker levels were markedly higher in the Alder Hey PICU cohort than in the ED cohorts for all diagnostic groups. Specific populations such as neonates and children with co-morbidity are also important to consider for validation studies. Following this strategy, it will become apparent in which groups of patients a potential new biomarker might or might not perform satisfactorily. This framework could easily be extended to account for non-viral and non-bacterial causes of febrile illness as well, as is currently being explored in the Diagnosis and Management of Febrile Illness using RNA Personalized Molecular Signature Diagnosis (DIAMONDS) study (65). The algorithm could be modified for adult studies, but would need to be validated using case biomarker studies from adults.

## Clinical Translation of Biomarkers and Future Research

Following satisfactory discovery and validation stages, the performance of a potential biomarker needs to be studied against clinically meaningful and patient-centered endpoints. Until now, only few high-quality randomized trials have evaluated this in the pediatric population. As one example, the Neopins study showed that PCT could successfully be used to shorten the duration of antibiotics in suspected early onset sepsis in neonates (66). Similarly, Baer et al. showed that the duration of antibiotic treatment could be guided by PCT in a pediatric ED setting (67). The UK BATCH *(“Biomarker-guided duration of Antibiotic Treatment in Children Hospitalized with confirmed or suspected bacterial infection*”) trial is currently recruiting patients, aiming to use PCT for guiding the duration of antibiotics in hospitalized children with an acute infection (68). Other studies showed limited impact of using biomarkers on the management of children with acute infections (69, 70). Successful clinical implementation of a biomarker is complex and multifactorial (71), as was shown in a trial implementing rapid diagnostics for malaria, in which physicians did not use the result to guide treatment, despite good diagnostic accuracy (72). Next, given the significant cost associated with diagnostic uncertainty in childhood febrile illness, diagnostic advances increasing the confidence to withhold antibiotics may yield considerable efficiency gains, especially in the sub-groups where the perceived risks of failing to identify potentially life-threatening bacterial infections are greatest (11). It is therefore imperative that future randomized trials of biomarkers include comprehensive cost-effectiveness analysis. In addition, future studies will need to focus on combinations of biomarkers that ideally include markers of both viral and bacterial infections and of other febrile illnesses including inflammatory disease (17, 63, 73). Throughout, we discussed biomarkers in blood, but future studies should additionally consider the optimal type of biosample for a biomarker, as some have marked improved diagnostic performance in sterile fluids such as cerebral spinal fluid (74). Lastly, establishing the likelihood of viral or bacterial disease in febrile children, as suggested in this manuscript, does not always relate to the severity of disease. Emerging evidence is providing more insight into the role of clinical signs and symptoms in predicting the severity of childhood illness (75, 76). Future studies should therefore combine new biomarkers with existing validated clinical prediction models with an aim to predict both severity and etiology of childhood febrile illness (77).

## Conclusion

The absence of a perfect reference standard for biomarker studies in serious bacterial infections has hindered translation of biomarker studies into clinical practice. Our proposed new algorithm provides a framework for phenotyping children with infections based on the trends in the different biomarkers in relation to the certainty of the diagnosis of either bacterial or viral categories. The findings from our independent biomarker validation studies suggest that the algorithm also aligns well with the host response and could provide mechanistic insights for those with uncertain diagnoses. To utilize the full potential of -omics driven biomarkers discovery studies, it will be essential to reach agreement on the best outcome reference standard in future studies, and we propose our diagnostic phenotyping algorithm as the best possible way to do so at present.

## PPI and Stakeholders' Statement

Patient representatives were involved in all facets of the PERFORM study involving study design, data collection and patient recruitment and presentation and dissemination of results, during the entirety of study period. The PERFORM consortium, made up of 18 organizations from 10 different countries, actively engages with national and European wide policymakers and stakeholders, to maximize the project's reach and impact.

## Data Availability Statement

The raw data supporting the conclusions of this article will be made available by the authors, without undue reservation.

## Ethics Statement

Patients were recruited with full institutional ethical approval, and informed consent was obtained from all included patients at the time of recruitment. The St. Mary's hospital cohort study was reviewed and approved by the St Mary's Research Ethics Committee (REC 09/H0712/58). For the Alder Hey ED and PICU cohorts approval for the study was granted by the Greater Manchester West Research Ethics Committee (10/H1014/53, 10/H1014/52) and by the Alder Hey Children's Hospital R&D department. The Maastad ED and Erasmus ED studies were approved by the institutional ethical committees of the Erasmus MC (MEC-2007-066) and the Maasstad Hospital (2010/64), respectively.

## Author Contributions

EC, JH, RN, HM, RO, CC-P, UB, IE, ME, MF, RG, BK, EL, IM, SP, FM-T, MP, SR, IC, LS, FS, MT, SY, DZ, WZ, ML, AC, MK, TD, TK, CF, EU, and VW: conception of the study and revision of manuscript. RN (first draft), EC, RO, JH, HM, and CC-P: drafting of manuscript. RN, EC, and JH: literature review. Data collection for the validation cohort studies: HM, RO, and RN were responsible for and supervised data collection for the Maasstad ED and Erasmus ED cohorts. JH and CC-P for the St. Mary's hospital cohort. EC for the Alder Hey ED and PICU cohorts. RN, RO, EC, CC-P, HM, and JH: analysis of the validation cohort studies and interpretation of results. Figures were constructed by RN (first versions), RO, EC, CC-P, HM, and JH. No medical writer or editor was involved in the writing of this manuscript. RN and EC confirm that they had full access to all the data in the study and are responsible for the decision to submit for publication. All authors have seen this version of the manuscript and agreed with it to be submitted.

## Conflict of Interest

CF is affiliated with Micropathology Ltd., an Independent Rapid Diagnosis & Biomedical Research Company. Micropathology Ltd. provides a clinically supported service for the rapid diagnosis and management of infectious and genetic disease. It is a formal partner of the PERFORM research consortium. The remaining authors declare that the research was conducted in the absence of any commercial or financial relationships that could be construed as a potential conflict of interest.

## Publisher's Note

All claims expressed in this article are solely those of the authors and do not necessarily represent those of their affiliated organizations, or those of the publisher, the editors and the reviewers. Any product that may be evaluated in this article, or claim that may be made by its manufacturer, is not guaranteed or endorsed by the publisher.
