# Supplementary material for: A Novel Framework for Phenotyping Children With Suspected or Confirmed Infection for Future Biomarker Studies
Source: Front Pediatr. 2021 Jul 28;9:688272. doi: 10.3389/fped.2021.688272 (PMC8356564; doi:10.3389/fped.2021.688272)
Supplement: Supplementary file 1 [file Table_1.docx]

# Appendix A. Overview of serious bacterial infections and the varying reference standards used in diagnostic accuracy studies

|  |  |  | **Type of sample** | **Definition of Positive test result** | **Example of clinical diagnosis** |
| --- | --- | --- | --- | --- | --- |
| **Serious infections** | **Serious bacterial infections (SBI) ^** | **Invasive bacterial infections** | Blood culture | Single bacterial pathogen isolated | Bacteraemia, sepsis |
|  |  |  | CSF culture | Single bacterial pathogen isolated (+- CSF pleocytosis) | Bacterial meningitis |
|  |  |  | Urine culture | Single bacterial pathogen isolated, monoculture; (+- urine microscopy and/or urinalysis) | Urinary tract infection, pyelonephritis |
|  |  |  | Blood PCR | Single bacterial pathogen PCR positive | Bacteraemia, sepsis |
|  |  |  | CSF PCR | Single bacterial pathogen PCR positive | Bacterial meningitis |
|  |  |  | Skin swab culture | Single bacterial pathogen isolated | Bacterial soft tissue infection.eg: Staphylococcal/Streptococcal infection |
|  |  |  | Throat swab culture | Single bacterial pathogen isolated | Group A Streptococcal infection, scarlet fever |
|  |  |  | Stools culture | Single bacterial pathogen isolated | Bacterial gastro-enteritis, e.g. Salmonella, Shigella |
|  |  |  | Sputum culture | Single bacterial pathogen isolated | Bacterial upper or lower respiratory tract infection |
|  |  |  | Broncho-alveolar lavage culture | Single bacterial pathogen isolated | Bacterial pneumonia |
|  |  |  | Wound swab culture | Single bacterial pathogen isolated | Bacterial soft tissue infection |
|  |  |  | Bacterial cultures other | Single bacterial pathogen isolated; e.g. from drained fluid form chest, abscess, or joint | Septic arthritis, bacterial empyema, abscess |
|  |  |  | Bacterial PCR other | Single bacterial pathogen PCR positive | Pertussis |
|  |  |  | Chest X ray | Evidence of lobar pneumonia, i.e. focal consolidation, or effusion, or empyema | Bacterial pneumonia |
|  |  |  | USS, CT, MRI, DMSA | Evidence of serious bacterial infection | abscess, preseptal / orbital cellulitis, mastoiditis, appendicitis, peritonitis, deep tissue infection, transverse myelitis/discitis, osteomyelitis; support diagnosis of UTI / pyelonephritis |
|  |  |  | Clinical diagnosis | In the absence of any diagnostics | Abscess, cellulitis, scarlet fever |
|  |  |  | Serology | Positive for acute bacterial infection (IgG, IgM) | Mycoplasma infection |
|  |  |  | Other | Positive urine antigen | Legionella; Pneumococcal infection (>5 years) |
|  | **Other or atypical (non-viral) serious infections** | | Histology |  | Appendicitis |
|  |  |  | Clinical criteria / consensus / echo |  | Kawasaki |
|  |  |  | Chest XR / IGRA / Mantoux / other / consensus |  | TB |
|  |  |  | CSF pleocytosis with predominant polymorphs | No pathogen identified, with CSF obtained post-antibiotics | Sterile or aseptic meningitis |
|  |  |  | Blood | Serology or PCR | Rickettsiosis |
|  |  |  | Fungus isolated from (non-) sterile site | Culture or PCR | Invasive or non-invasive fungal infection |
|  |  |  | Blood | Parasitaemia and microscopy | Malaria |
|  | **Serious viral infections** |  | Any sample type | PCR or culture | Confirmed causative viral pathogen in serious illness in need for hospital admission and (intensive) supportive care |

^ as used to define Serious Bacterial Infections in the five independent validation cohorts; with more detail available in the original publications. Diagnostic results from complete disease episode interpreted in clinical context by expert consensus.
