# Supplementary material for: A Novel Framework for Phenotyping Children With Suspected or Confirmed Infection for Future Biomarker Studies
Source: Front Pediatr. 2021 Jul 28;9:688272. doi: 10.3389/fped.2021.688272 (PMC8356564; doi:10.3389/fped.2021.688272)
Supplement: Supplementary file 2 [file Table_2.docx]

# Appendix B. Correlation between C-Reactive Protein and markers of bacterial infection

|  | **Pearson correlation coefficient**  **(95% CI)** | **p-value** |
| --- | --- | --- |
| **Alder Hey ED cohort** |  |  |
| NGAL (ng/mL) | 0·37  (0·32–0·42) | <0.001 |
| Resistin (ng/mL) | 0·14  (0·09–0·20) | <0.001 |
| PCT (µg/L) | 0·37  (0·32–0·42) | <0.001 |
| **Alder Hey PICU cohort** |  |  |
| NGAL (ng/mL) | 0·23  (0·08–0·37) | 0.002 |
| Resistin (ng/mL) | 0·21  (0·06–0·35) | 0.006 |
| PCT (µg/L) | 0·25  (0·15–0·35) | <0.001 |
| **Erasmus ED cohort** |  |  |
| PCT (µg/L) | 0·36  (0·30–0·43) | <0.001 |
| **Maasstad ED cohort** |  |  |
| PCT (µg/L) | 0·41  (0·32–0·49) | <0.001 |
| **St. Mary’s hospital cohort** |  |  |
| NGAL (ng/mL) | 0·40  (0·26 – 0·53) | <0.001 |
