# Supplementary material for: A Novel Framework for Phenotyping Children With Suspected or Confirmed Infection for Future Biomarker Studies
Source: Front Pediatr. 2021 Jul 28;9:688272. doi: 10.3389/fped.2021.688272 (PMC8356564; doi:10.3389/fped.2021.688272)
Supplement: Supplementary file 3 [file Data_Sheet_1.docx]

*The PERFORM consortium (*<https://www.perform2020.org/>) *is composed by:*

**PARTNER: Imperial College, London, UK**

Imperial College: Faculty of Medicine

Michael Levin^1^ (grant application, PERFORM Coordinator, CI)

Aubrey Cunnington^1^ (grant application, clinical translation), Tisham De^1^ (work package lead), Jethro A Herberg^1^ (grant application, PI), Myrsini Kaforou^1^ (grant application, bioinformatics), Victoria J Wright^1^ (grant application, scientific coordination)

Imperial College: consortium members^1^ (in alphabetical order)

Lucas Baumard, Lachlan Coin, Giselle D’Souza, Rachel Galassini, Dominic Habgood-Coote, Shea Hamilton, Clive Hoggart, Sara Hourmat, Heather Jackson, Naomi Lin, Ian Maconochie^4^, Stephanie Menikou, Samuel Nichols, Ruud Nijman, Ivonne Pena Paz, Priyen Shah, Clare Wilson, Ching-Fen Shen

Imperial College: Faculty of Engineering^2^

Molly Stevens (co-investigator), Eunjung Kim, Nayoung Kim, Benjamin Pierce

Imperial College Healthcare NHS Trust^3^: clinical recruitment (alphabetical order)

Ladan Ali, Sarah Darnell, Rikke Jorgensen, Sobia Mustafa, Salina Persand

PERFORM UK Clinical Network: Clinical recruitment at Brighton and Sussex University Hospitals^5^

Katy Fidler (principal investigator), Julia Dudley (Clinical Research Registrar), Vivien Richmond (research nurse), Emma Tavliavini (research nurse)

Author Affiliations:

1. Section of Paediatric Infectious Disease, Wright-Fleming Institute, Norfolk Place, London W2 1PG, UK
2. Department of Materials, Royal School of Mines, Prince Consort Rd, London SW7 2AZ, UK
3. Children’s Clinical Research Unit, St Mary’s Hospital, Praed Street, London W2 1NY, UK
4. Paediatric Emergency Dept, St Mary’s Hospital, Praed Street, London W2 1NY, UK
5. Royal Alexandra Children’s Hospital, Brighton, BN2 5BE, UK.

**PARTNER: SERGAS, Santiago de Compostela, Spain**

Principal Investigators

Federico Martinón-Torres^1^, Antonio Salas^1,2^

GENVIP RESEARCH GROUP (in alphabetical order)

Fernando Álvez González^1^, Cristina Balo Farto^1^, Ruth Barral-Arca^1,2^, María Barreiro Castro^1^, Xabier Bello^1,2^, Mirian Ben García^1^, Sandra Carnota^1^, Miriam Cebey-López^1^, María José Curras-Tuala^1,2^, Carlos Durán Suárez^1^, Luisa García Vicente^1^, Alberto Gómez-Carballa^1,2^, Jose Gómez Rial^1^, Pilar Leboráns Iglesias^1^, Federico Martinón-Torres^1^, Nazareth Martinón-Torres^1^, José María Martinón Sánchez^1^, Belén Mosquera Pérez^1^, Jacobo Pardo-Seco^1,2^, Lidia Piñeiro Rodríguez^1^, Sara Pischedda^1,2^, Sara Rey Vázquez^1^, Irene Rivero Calle^1^, Carmen Rodríguez-Tenreiro^1^, Lorenzo Redondo-Collazo^1^, Miguel Sadiki Ora^1^, Antonio Salas^1,2^, Sonia Serén Fernández^1^, Cristina Serén Trasorras^1^, Marisol Vilas Iglesias^1^

Author Affiliations:

1. Translational Pediatrics and Infectious Diseases, Pediatrics Department, Hospital Clínico Universitario de Santiago, Santiago de Compostela, Spain, and GENVIP Research Group (www.genvip.org), Instituto de Investigación Sanitaria de Santiago, Universidad de Santiago de Compostela, Galicia, Spain.
2. Unidade de Xenética, Departamento de Anatomía Patolóxica e Ciencias Forenses, Instituto de Ciencias Forenses, Facultade de Medicina, Universidade de Santiago de Compostela, and GenPop Research Group, Instituto de Investigaciones Sanitarias (IDIS), Hospital Clínico Universitario de Santiago, Galicia, Spain
3. Fundación Pública Galega de Medicina Xenómica, Servizo Galego de Saúde (SERGAS), Instituto de Investigaciones Sanitarias (IDIS), and Grupo de Medicina Xenómica, Centro de Investigación Biomédica en Red de Enfermedades Raras (CIBERER), Universidade de Santiago de Compostela (USC), Santiago de Compostela, Spain

**PARTNER: Riga Stradins university, Riga, Latvia**

Principal Investigator

Dace Zavadska^1,2^

Other RSU group authors (in alphabetical order)

Anda Balode^1,2^, Arta Bārzdiņa^1,2^, Dārta Deksne^1,2^, Dace Gardovska^1,2^, Dagne Grāvele^2^, Ilze Grope^1,2^, Anija Meiere^1,2^, Ieva Nokalna^1,2^, Jana Pavāre^1,2^, Zanda Pučuka^1,2^, Katrīna Selecka^1,2^, Aleksandra Sidorova^1,2^, Dace Svile^2^, Urzula Nora Urbāne^1,2^.

Author Affiliations:

1. Riga Stradins university, Riga, Latvia.
2. Children clinical university hospital, Riga, Latvia.

**PARTNER: Medical Research Council Unit The Gambia (MRCG) at LSHTM Partner**

Principal Investigator

Effua Usuf

Additional Investigators

Kalifa Bojang, Syed M. A. Zaman, Fatou Secka, Suzanne Anderson, Anna Rocalsatou Sarr, Momodou Saidykhan, Saffiatou Darboe, Samba Ceesay, Umberto D’alessandro

Author Affiliations:

Medical Research Council Unit The Gambia at LSHTM.

P O Box 273, Fajara, The Gambia

**PARTNER: Erasmus MC - Sophia Children’s Hospital, Rotterdam, The Netherlands**

Principal Investigator

Henriëtte A. Moll¹

Research group

Dorine M. Borensztajn¹, Nienke N. Hagedoorn¹, Chantal Tan¹, Clementien L. Vermont², Joany Zachariasse¹

Additional investigator

Wim A Dik^3^

Author Affiliations:

1. Erasmus MC-Sophia Children’s Hospital, Department of General Paediatrics, Rotterdam, the Netherlands
2. Erasmus MC-Sophia Children’s Hospital, Department of Paediatric Infectious Diseases & Immunology, Rotterdam, the Netherlands
3. Erasmus MC, Department of immunology, Rotterdam, the Netherlands

**PARTNER: Swiss Pediatric Sepsis Study**

Principal Investigators

Philipp Agyeman^1^, Luregn J Schlapbach^1,2,3^, Eric Giannoni^4,5^, Martin Stocker^6^, Klara M Posfay-Barbe^7^, Ulrich Heininger^8^, Sara Bernhard-Stirnemann^9^, Anita Niederer-Loher^10^, Christian Kahlert^10^, Giancarlo Natalucci^11^, Christa Relly^12^, Thomas Riedel^13^, Christoph Aebi^1^, Christoph Berger^12^ *for the Swiss Pediatric Sepsis Study*

Author Affiliations:

1. Department of Pediatrics, Inselspital, Bern University Hospital, University of Bern, Switzerland
2. Paediatric Critical Care Research Group, Mater Research Institute, University of Queensland, Brisbane, Australia
3. Paediatric Intensive Care Unit, Lady Cilento Children’s Hospital, Children’s Health Queensland, Brisbane, Australia
4. Clinic of Neonatology, Department Mother-Woman-Child, Lausanne University Hospital and University of Lausanne, Switzerland
5. Infectious Diseases Service, Lausanne University Hospital, Lausanne, Switzerland
6. Department of Pediatrics, Children’s Hospital Lucerne, Lucerne, Switzerland
7. Pediatric Infectious Diseases Unit, Children’s Hospital of Geneva, University Hospitals of Geneva, Geneva, Switzerland
8. Infectious Diseases and Vaccinology, University of Basel Children’s Hospital, Basel, Switzerland
9. Children’s Hospital Aarau, Aarau, Switzerland
10. Division of Infectious Diseases and Hospital Epidemiology, Children’s Hospital of Eastern Switzerland St. Gallen, St. Gallen, Switzerland
11. Department of Neonatology, University Hospital Zurich, Zurich, Switzerland
12. Division of Infectious Diseases and Hospital Epidemiology, and Children’s Research Center, University Children’s Hospital Zurich, Switzerland
13. Children’s Hospital Chur, Chur, Switzerland

**PARTNER: University of Liverpool, UK**

Principal Investigators

Enitan D Carrol^1,2,3^, Stéphane Paulus^1^

Research Group (in alphabetical order)

Elizabeth Cocklin^1^, Rebecca Jennings^4^, Joanne Johnston^4^, Simon Leigh^1^, Karen Newall^4^, Sam T Romaine^1^

Author Affiliations:

1. Department of Clinical Infection, Microbiology and Immunology, University of Liverpool Institute of Infection and Global Health , Liverpool, England
2. Alder Hey Children’s Hospital, Department of Infectious Diseases, Eaton Road, Liverpool, L12 2AP
3. Liverpool Health Partners, 1st Floor, Liverpool Science Park, 131 Mount Pleasant, Liverpool, L3 5TF
4. Alder Hey Children’s Hospital, Clinical Research Business Unit, Eaton Road, Liverpool, L12 2AP

**PARTNER: National and Kapodistrian University of Athens (NKUA), Greece**

Principal investigator

Maria Tsolia (all activities)

Investigator/Research fellow

Irini Eleftheriou (all activities)

Additional investigators

Maria Tambouratzi (recruitment), Antonis Marmarinos (Lab, Quality Manager), Marietta Xagorari (Lab), Kelly Syggelou

Author Affiliations:

2nd Department of Pediatrics, National and Kapodistrian University of Athens,

“P. and A. Kyriakou” Children’s Hospital

Thivon and Levadias

Goudi, Athens

**PARTNER : Micropathology Ltd, Warwick, UK**

Colin Fink, Marie Voice, Leo Calvo-Bado

**PARTNER : Medical University of Graz (MUG), Austria**

Principal Investigator

Werner Zenz^1^ (all activities)

Co-investigators (in alphabetical order)

Benno Kohlmaier^1^ (all activities), Nina A. Schweintzger^1^ (all activities), Manfred G. Sagmeister^1^ (study design, consortium wide sample management)

Research team

Daniela S. Kohlfürst^1^ (study design), Christoph Zurl^1^ (BIVA PIC), Alexander Binder^1^ (grant application)

Recruitment team, data managers, (in alphabetical order)

Susanne Hösele^1^, Manuel Leitner^1^, Lena Pölz^1^, Glorija Rajic^1^

Clinical recruitment partners (in alphabetical order)

Sebastian Bauchinger^1^, Hinrich Baumgart^6^, Martin Benesch^3^, Astrid Ceolotto^1^, Ernst Eber^2^, Siegfried Gallistl^1^, Gunther Gores^5^, Harald Haidl^1^, Almuthe Hauer^1^, Christa Hude^1^, Markus Keldorfer^5^, Larissa Krenn^4^, Heidemarie Pilch^5^, Andreas Pfleger^2^, Klaus Pfurtscheller^4^, Gudrun Nordberg^5^, Tobias Niedrist^8^, Siegfried Rödl^4^, Andrea Skrabl-Baumgartner^1^, Matthias Sperl^7^, Laura Stampfer^5^, Volker Strenger^3^, Holger Till^6^, Andreas Trobisch^5^, Sabine Löffler^5^

Author Affiliations:

1. Medical University of Graz, Department of General Paediatrics, Austria
2. Medical University of Graz, Department of Paediatric Pulmonology, Austria
3. Medical University of Graz, Department of Paediatric Hemato-oncoloy, Austria
4. Medical University of Graz, Paediatric Intensive Care Unit, Austria
5. Medical University Graz, University Clinic of Paediatrics and Adolescent Medicine, Austria
6. Medical University of Graz, Department of Paediatric and Adolescent Surgery, Austria
7. Medical University Graz, Department of Pediatric Orthopedics, Austria
8. Medical University Graz, Clinical Institute of Medical and Chemical Laboratory Diagnostics, Austria

**PARTNER: London School of Hygiene and Tropical Medicine, UK**

Principal Investigator

Shunmay Yeung^1,2 3^

Research Group

Martin Hibberd^1^, Juan Emmanuel Dewez^1^, David Bath^2^, Alec Miners^2^, Ruud Nijman^3,^ Catherine Wedderburn^1^, Anne Meierford^1^, Baptiste Leurent^4^

Author Affiliations:

1. Faculty of Infectious and Tropical Disease, London School of Hygiene and Tropical Medicine, London, UK
2. Faculty of Public Health and Policy, London School of Hygiene and Tropical Medicine, London, UK
3. Department of Paediatrics, St. Mary’s Hospital Imperial College Hospital, London, UK
4. Faculty of Epidemiology and Population Health, London School of Hygiene and Tropical Medicine, London, UK

**PARTNER : Radboud University Medical Center (RUMC),** **Nijmegen,** **The Netherlands**

Principal Investigators:

Ronald de Groot^1^, Michiel van der Flier^1,2,3^, Marien I. de Jonge^1^

Co-investigators Radboud University Medical Center (in alphabetical order)

Koen van Aerde^1,2^, Wynand Alkema^1^, Bryan van den Broek^1^, Jolein Gloerich^1^, Alain J. van Gool^1^, Stefanie Henriet^1,2^, Martijn Huijnen^1^, Ria Philipsen^1^, Esther Willems^1^

Investigators PeDBIG PERFORM DUTCH CLINICAL NETWORK (in alphabetical order)

G.P.J.M. Gerrits^8^, M. van Leur^8^, J. Heidema ^4^, L. de Haan^1,2^, C.J. Miedema ^5^, C. Neeleman ^1^, C.C. Obihara ^6^, G.A. Tramper-Stranders^7^

Author Affiliations:

1. Radboud University Medical Center, Nijmegen, The Netherlands
2. Amalia Children’s Hospital, Nijmegen, The Netherlands
3. Wilhelmina Children’s Hospital, University Medical Center Utrecht, Utrecht, The Netherlands
4. St. Antonius Hospital, Nieuwegein, The Netherlands
5. Catharina Hospital, Eindhoven, The Netherlands
6. ETZ Elisabeth, Tilburg, The Netherlands
7. Franciscus Gasthuis, Rotterdam, The Netherlands
8. Canisius Wilhelmina Hospital, Nijmegen, The Netherlands

**PARTNER : University of Oxford, UK**

Principal Investigators

Andrew J. Pollard^1,2^, Rama Kandasamy^1,2^, Stéphane Paulus ^1,2^

Additional Investigators

Michael J. Carter^1,2^, Daniel O'Connor^1,2^, Sagida Bibi^1,2^, Dominic F. Kelly^1,2^, Meeru Gurung^3^, Stephen Thorson^3^, Imran Ansari^3^, David R. Murdoch^4^, Shrijana Shrestha^3^.

Author Affiliations:

1. Oxford Vaccine Group, Department of Paediatrics, University of Oxford, Oxford, United Kingdom.
2. NIHR Oxford Biomedical Research Centre, Oxford, United Kingdom.
3. Paediatric Research Unit, Patan Academy of Health Sciences, Kathmandu, Nepal.
4. Department of Pathology, University of Otago, Christchurch, New Zealand.

**PARTNER: Newcastle University, Newcastle upon Tyne, UK**

Principal Investigator

Marieke Emonts ^1,2,3^ (all activities)

Co-investigators

Emma Lim^2,3,7^ (all activities), Lucille Valentine^4^

Recruitment team (alphabetical), data-managers, and GNCH Research unit

Karen Allen^5^, Kathryn Bell^5^, Adora Chan^5^, Stephen Crulley^5^, Kirsty Devine^5^, Daniel Fabian^5^, Sharon King^5^, Paul McAlinden^5^, Sam McDonald^5^, Anne McDonnell2,^5^, Ailsa Pickering^2,5^, Evelyn Thomson^5^, Amanda Wood^5^, Diane Wallia^5^, Phil Woodsford^5^,

Sample processing: Frances Baxter^5^, Ashley Bell^5^, Mathew Rhodes^5^

PICU recruitment

Rachel Agbeko^8^, Christine Mackerness^8^

Students MOFICHE

Bryan Baas^2^, Lieke Kloosterhuis^2^, Wilma Oosthoek^2^

Students/medical staff PERFORM

Tasnim Arif^6^, Joshua Bennet^2^, Kalvin Collings^2^, Ilona van der Giessen^2^, Alex Martin^2^, Aqeela Rashid^6^, Emily Rowlands^2^, Gabriella de Vries^2^, Fabian van der Velden^2^

Engagement work/ethics/cost effectiveness

Mike Martin^9^, Ravi Mistry^2^, Lucille Valentine^4^

Author Affiliations:

1. Translational and Clinical Research Institute, Newcastle University, Newcastle upon Tyne UK
2. Great North Children’s Hospital, Paediatric Immunology, Infectious Diseases & Allergy, Newcastle upon Tyne Hospitals NHS Foundation Trust, Newcastle upon Tyne, United Kingdom.
3. NIHR Newcastle Biomedical Research Centre based at Newcastle upon Tyne Hospitals NHS Trust and Newcastle University, Westgate Rd, Newcastle upon Tyne NE4 5PL, United Kingdom
4. Newcastle University Business School, Centre for Knowledge, Innovation, Technology and Enterprise (KITE), Newcastle upon Tyne, United Kingdom
5. Great North Children’s Hospital, Research Unit, Newcastle upon Tyne Hospitals NHS Foundation Trust, Newcastle upon Tyne, United Kingdom.
6. Great North Children’s Hospital, Paediatric Oncology, Newcastle upon Tyne Hospitals NHS Foundation Trust, Newcastle upon Tyne, United Kingdom.
7. Population Health Sciences Institute, Newcastle University, Newcastle upon Tyne, UK
8. Great North Children’s Hospital, Paediatric Intensive Care Unit, Newcastle upon Tyne Hospitals NHS Foundation Trust, Newcastle upon Tyne, United Kingdom.
9. Northumbria University, Newcastle upon Tyne, United Kingdom

**PARTNER : University Medical Centre Ljubljana, Slovenia**

Principal Investigator

Marko Pokorn^1,2,3^ MD, PhD

Research Group

Mojca Kolnik^1^ MD, Katarina Vincek^1^ MD, Tina Plankar Srovin^1^ MD, PhD, Natalija Bahovec^1^ MD, Petra Prunk^1^ MD, Veronika Osterman^1^ MD, Tanja Avramoska^1^ MD

Author Affiliations:

1. Department of Infectious Diseases, University Medical Centre Ljubljana, Japljeva 2, SI-1525 Ljubljana, Slovenia
2. University Childrens' Hospital, University Medical Centre Ljubljana, Ljubljana, Slovenia
3. Department of Infectious Diseases and Epidemiology, Faculty of Medicine, University of Ljubljana, Slovenia

**PARTNER : Academic Medical Hospital & Sanquin Research Institute, Amsterdam, NL**

Principal Investigator

Taco Kuijpers ^1,2^

Co-investigators

Ilse Jongerius ^2^

Recruitment team (EUCLIDS, PERFORM)

J.M. van den Berg^1^, D. Schonenberg^1^, A.M. Barendregt^1^, D. Pajkrt^1^, M. van der Kuip^1,3^, A.M. van Furth^1,3^

Students PERFORM

Evelien Sprenkeler ^2^, Judith Zandstra ^2^

Technical support PERFORM

G. van Mierlo ^2^, J. Geissler ^2^

Author Affiliations:

1. Amsterdam University Medical Center (Amsterdam UMC), location Academic Medical Center (AMC), Dept of Pediatric Immunology, Rheumatology and Infectious Diseases, University of Amsterdam, Amsterdam, the Netherlands
2. Sanquin Research Institute, & Landsteiner Laboratory at the AMC, University of Amsterdam, Amsterdam, the Netherlands.
3. Amsterdam University Medical Center (Amsterdam UMC), location Vrije Universiteit Medical Center (VUMC), Dept of Pediatric Infectious Diseases and Immunology, Free University (VU), Amsterdam, the Netherlands (former affiliation)

**PARTNER: National Cheng Kung University Hospital, Taiwan**

Ching-Fen Shen (Principal Investigator); Ching-Chuan Liu (Co-investigator); Shih-Min Wang (Co-investigator), funded by the Center of Clinical Medicine Research, National Cheng Kung University

**PARTNER: Ludwig Maximilians University Munich, Germany**

Principal Investigator:

Ulrich von Both^1,2^

Research group:

Laura Kolberg¹, Manuela Zwerenz¹, Judith Buschbeck¹

Clinical recruitment partners (in alphabetical order):

Christoph Bidlingmaier^3^, Vera Binder^4^, Katharina Danhauser^5^, Nikolaus Haas^10^, Matthias Griese^6^, Tobias Feuchtinger^4^, Julia Keil^9^, Matthias Kappler^6^, Eberhard Lurz^7^, Georg Muench^8^, Karl Reiter^9^, Carola Schoen^9^

Author Affiliations:

1. Div. Paediatric Infectious Diseases, Hauner Children’s Hospital, University Hospital, Ludwig Maximilians University (LMU), Munich, Germany
2. German Center for Infection Research (DZIF), Partner Site Munich, Munich, Germany
3. Div. of General Paediatrics, Hauner Children’s Hospital, University Hospital, Ludwig Maximilians University (LMU), Munich, Germany
4. Div. Paediatric Haematology & Oncology, Hauner Children’s Hospital, University Hospital, Ludwig Maximilians University (LMU), Munich, Germany
5. Div. of Paediatric Rheumatology, Hauner Children’s Hospital, University Hospital, Ludwig Maximilians University (LMU), Munich, Germany
6. Div. of Paediatric Pulmonology, Hauner Children’s Hospital, University Hospital, Ludwig Maximilians University (LMU), Munich, Germany
7. Div. of Paediatric Gastroenterology, Hauner Children’s Hospital, University Hospital, Ludwig Maximilians University (LMU), Munich, Germany
8. Neonatal Intensive Care Unit, Hauner Children’s Hospital, University Hospital, Ludwig Maximilians University (LMU), Munich, Germany
9. Paediatric Intensive Care Unit Hauner Children’s Hospital, University Hospital, Ludwig Maximilians University (LMU), Munich, Germany
10. Department Pediatric Cardiology and Pediatric Intensive Care, University Hospital, Ludwig Maximilians University (LMU), Munich, Germany

**PARTNER: bioMérieux, France**

Principal Investigator:

François Mallet^1,2, 3^

Research Group:

Karen Brengel-Pesce^1,2,3^, Alexandre Pachot^1^, Marine Mommert^1,2^

1. Open Innovation & Partnerships (OIP), bioMérieux S.A., Marcy l'Etoile, France
2. Joint research unit Hospice Civils de Lyon - bioMérieux, Centre Hospitalier Lyon Sud, 165 Chemin du Grand Revoyet, 69310 Pierre-Bénite, France
3. EA 7426 Pathophysiology of Injury-induced Immunosuppression, University of Lyon1-Hospices Civils de Lyon-bioMérieux, Hôpital Edouard Herriot, 5 Place d’Arsonval, 69437 Lyon Cedex 3, France
